# Expression and characterization of pantothenate energy‐coupling factor transporters as an anti‐infective drug target

**DOI:** 10.1002/pro.5195

**Published:** 2024-10-29

**Authors:** Atanaz Shams, Spyridon Bousis, Eleonora Diamanti, Walid A. M. Elgaher, Lucie Zeimetz, Jörg Haupenthal, Dirk J. Slotboom, Anna K. H. Hirsch

**Affiliations:** ^1^ Helmholtz Institute for Pharmaceutical Research Saarland (HIPS) – Helmholtz Centre for Infection Research (HZI) Department of Drug Design and Optimization Saarbrücken Germany; ^2^ Saarland University Department of Pharmacy Saarbrücken Germany; ^3^ Stratingh Institute for Chemistry and Technology, Faculty of Science and Engineering University of Groningen Groningen The Netherlands; ^4^ Department of Pharmacy and Biotechnology Alma Mater Studiorum—Università di Bologna Bologna Italy; ^5^ Groningen Biomolecular Sciences and Biotechnology Institute University of Groningen Groningen The Netherlands

**Keywords:** antimicrobial resistance (AMR), B‐vitamins, energy‐coupling factor (ECF) transporters, *Galleria mellonella* infection model, pantothenate (vitamin B‐5), *Streptococcus pneumoniae*, surface plasmon resonance

## Abstract

This study investigates the potential of energy‐coupling factor (ECF) transporters as promising anti‐infective targets to combat antimicrobial resistance (AMR). ECF transporters, a subclass of ATP‐binding cassette (ABC) transporters, facilitate the uptake of B‐vitamins across bacterial membranes by utilizing ATP as an energy source. Vitamins are essential cofactors for bacterial metabolism and growth, and they can either be synthesized de novo or absorbed from the environment. These transporters are considered promising drug targets, underscoring the need for further research to harness their medicinal potential and develop selective inhibitors that block vitamin uptake in bacteria. Herein, we focused on the ECF transporter for pantothenate (vitamin B5) from *Streptococcus pneumoniae* and the ECF transporter for folate (vitamin B9) from *Lactobacillus delbrueckii* as a reference protein. We also included the energizing module for pantothenate along with both full transporter complexes. Initially, we transformed and purified the transporters, followed by an assessment of their thermal stability under various buffer composition, pH, and salt concentrations. Additionally, we monitored the melting temperature over six days to confirm their stability for further assays. We then measured the binding affinities of six ECF inhibitors using surface plasmon resonance (SPR) and evaluated their inhibitory effects through in vitro assays, including bacterial growth assay, whole‐cell uptake, and transport‐activity assays. After determining cytotoxicity in two human cell lines, we established an in vivo infection model using *Galleria mellonella* larvae to further validate our findings.

## 1 INTRODUCTION

Antimicrobial resistance (AMR) is a major global public health threat that endangers humans. It occurs when a microbe gains the ability to evade the effect of anti‐infectives (Murray et al., 2022). The World Health Organization (WHO) list of global‐priority, drug‐resistant pathogens in 2024 includes macrolide‐resistant Group A Streptococci, penicillin‐resistant Group B Streptococci, macrolide‐resistant *Streptococcus pneumoniae*, and *Haemophilus influenzae*, underscoring the urgent need to address their public health impacts, particularly for vulnerable populations in resource‐limited settings. This highlights the critical necessity to discover novel anti‐infective therapies, especially those with unprecedented modes of action.

The energy‐coupling factor (ECF) transporters are a subfamily of the ATP‐binding cassette (ABC) transporters that mediate the uptake of substrates such as B‐type vitamins, Ni^2+^, and Co^2+^ through the membrane of prokaryotes. Due to their specific role in the bacterial homeostasis, they might represent a new avenue to treat infections caused by bacteria that rely on these transporters for survival (Henderson et al., 1979; Rempel et al., 2019; Rodionov et al., 2009).

We selected *S. pneumoniae* as a suitable target pathogen to study the function of ECF transporters as it lacks the biosynthetic route for pantothenate (vitamin B‐5), biotin (vitamin B‐7), and folate (vitamin B‐9), and it depends on their uptake from the environment (Bousis et al., 2018). *S. pneumoniae* is a Gram‐positive pathogen that causes pneumonia and meningitis by targeting and colonizing the respiratory tract. Although many pneumococcal infections can be treated using antibiotics, mutated *S. pneumoniae* strains appear with resistance against current antibiotics (Cillóniz et al., 2018).

Vitamins are indispensable for the survival of bacteria, archaea as well as humans. Prokaryotes can synthesize vitamins from primary metabolites or conversely, they can be auxotrophic and rely on the uptake of vitamins from the environment (Bousis et al., 2018). In the latter case, bacteria cannot synthesize vitamins de novo or do not have access to their entire biosynthetic pathway (Jaehme & Slotboom, 2014).

Intrigued by the critical role the ECF transporters played in the uptake of B‐type vitamins, the focus of this work is directed toward the study of ECF transporter for pantothenate in *S. pneumoniae*.

**FIGURE 1:**
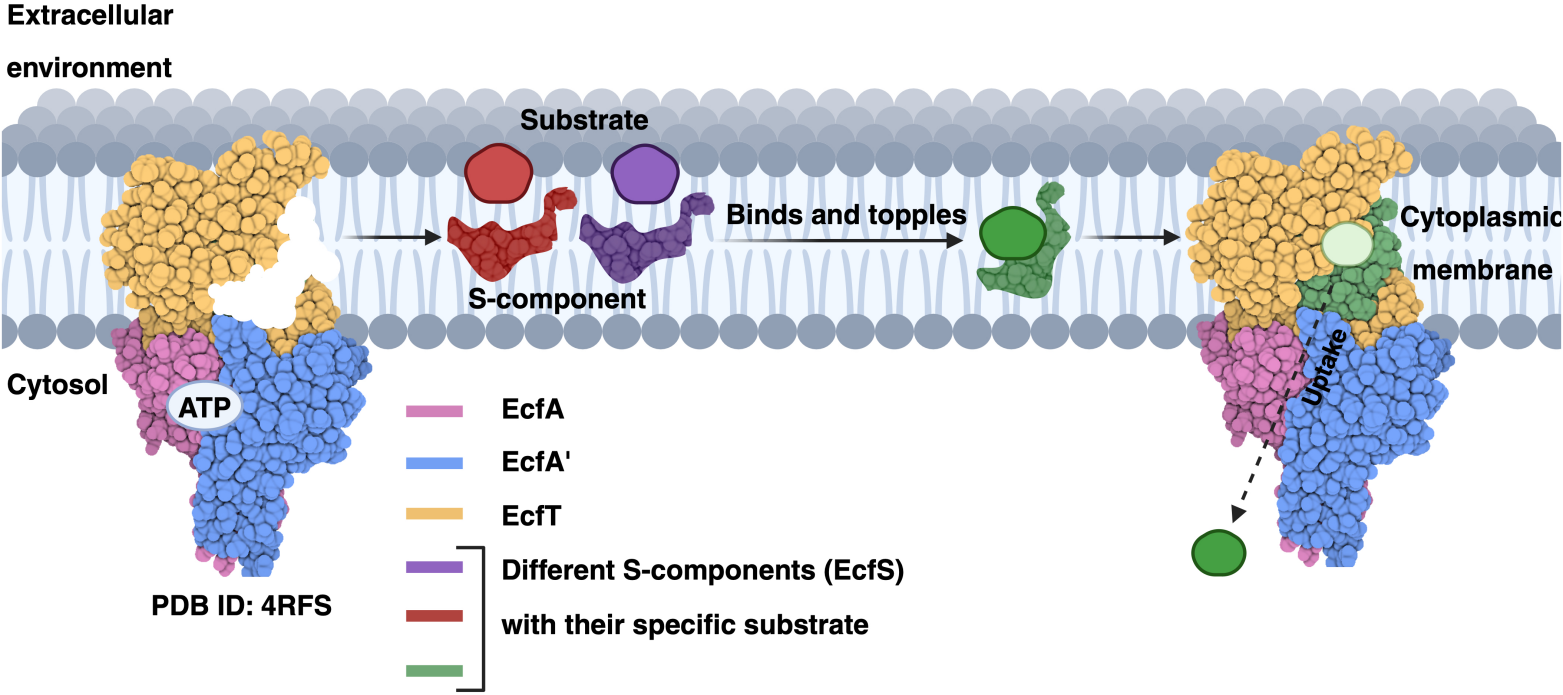
Schematic representation of the ECF transporters subunits and mechanism of transport. The ECF transporters use the integral‐membrane S proteins (EcfS) to bind to a substrate and transport it into the cytoplasm in an ATP‐dependent process (Zhang, 2013; Zhang et al., 2014). *Source*: Created with BioRender.com.

Structurally, ECF transporters contain four domains: two cytosolic ATPases named EcfA and EcfA′, a membrane‐embedded substrate‐binding protein known as EcfS or S‐component, and a transmembrane protein called EcfT that connects the two ATPases (EcfA and EcfA′) and the S‐component (Figure 1) (Zhang, 2013).

Two classifications are found for ECF transporters. In group I, each ECF module (EcfTAA′) interacts with its specific S‐component, and a single operon is responsible for the genes encoding all different domains. In group II, multiple S‐components compete for the same ECF module, engaging in shared competition. The genes encoding S‐components are distinct from the other three domains and are scattered across the chromosome (Slotboom, 2014).

To explore ECF transporters for pantothenate in *S. pneumoniae*, we expressed and isolated three proteins: (i) the *S. pneumoniae* full ECF transporter complex for pantothenate (ECF‐PanT) with PanT as the S‐component, (ii) the *S. pneumoniae* EcfTAA′ (ECF module), and (iii) the *L. delbrueckii* full ECF transporter complex for folic acid (ECF‐FolT2) as a comparative model. We evaluated the stability of ECF‐PanT and the ECF module using thermal shift assay (TSA) and determined the binding affinities and inhibitory activities of a series of compounds previously identified by our group (Bousis et al., 2021; Diamanti et al., 2023; Drost et al., 2022) using various biochemical and biophysical assays. Ultimately, we assessed the compounds in vivo against *S. pneumoniae* using *Galleria mellonella* larvae infection model.

This study serves as a proof‐of‐concept investigation aimed at exploring and validating the feasibility of our experimental approaches and hypotheses. By focusing on the purification and characterization of the ECF‐PanT transporter and its interactions with various inhibitors, we seek to establish a foundational understanding of these systems. The insights gained from this work are intended to guide further research and development in the field, particularly in the context of targeting ECF transporters for therapeutic applications.

## 2 RESULTS

### 2.1 Sequence alignment and protein homology determination

Comparative genome sequence analysis indicated that the energy‐coupling modules are conserved among bacteria (Rodionov et al., 2009). Previously, we carried out sequence alignment of the ECF modules across seven pathogens, including *Staphylococcus aureus*, *S. pneumoniae*, *Enterococcus faecium*, *Enterococcus faecalis*, *Clostridium tetani*, *Clostridium novyi*, and *Clostridium difficile*, highlighting the presence of highly conserved regions within the ECF modules. In particular, the ATP‐binding pocket and the interfaces between EcfA, EcfA′, and EcfT subunits, are quite conserved suggesting that these regions are critical for the transport function and could serve as potential drug targets (Bousis et al., 2018).

In this study, we further aligned the ECF‐PanT sequence from *S. pneumoniae* with three other ECF transporters, for which crystal structures have been determined, namely ECF‐FolT2 from *L. delbrueckii* (PDB: 5JSZ) (Swier et al., 2016), ECF‐PanT sequences from *L. delbrueckii* (PDB: 6ZG3) (Setyawati et al., 2020), and *Levilactobacillus brevis* (PDB: 4RFS) (Zhang et al., 2014) using the NCBI BLAST tool (Altschul et al., 1997, 2005). ECF‐FolT2 was used as protein model for comparison in our analysis (Swier et al., 2016). The results indicated sequence homology across the queries with >40% identity for *L. delbrueckii* ECF‐PanT *L. delbrueckii* ECF‐FolT2, and >36% identity was observed in *L. brevis* ECF‐PanT, in agreement with previous findings (Figure S1, Tables S1 and S2).

Consequently, we built a homology model of *S. pneumoniae* ECF‐PanT employing AlphaFold‐generated structures for the four ECF‐PanT domains (Jumper et al., 2021) and the structure of *L. delbrueckii* ECF‐FolT2 (PDB ID: 5JSZ) as a template for assembling the four protein subunits (Figure S2a). The homology model of *S. pneumonaie* ECF‐PanT revealed structural similarities with *L. delbrueckii* ECF‐FolT2 with an overall root mean square deviation (RMSD) of 3.25 Å. In particular, the two ATPase subunits (EcfA and EcfA′) showed RMSD values of 1.71 and 2.12 Å, respectively, which are consistent with their conserved functional roles, whereas high RMSD value was observed for the PanT component (5.12 Å) (Figure S2b), indicating potential structural divergence, possibly related to differences in substrate specificity. It is worth mentioning that high structural similarity were also observed between the *L. delbrueckii* ECF‐FolT2 and ECF‐PanT structures (Setyawati et al., 2020).

### 2.2 Stability determination

We investigated the stability of ECF‐PanT, ECF module, and ECF‐FolT2 proteins using the thermal shift assay (TSA). Furthermore, we used TSA to identify the buffer conditions that optimize protein stability for binding assays, specifically surface plasmon resonance (SPR). The optimal concentrations of GloMelt and ROX dyes in complex with ECF‐PanT in TSA were found to be 1× and 0.5 μM, respectively. Under these conditions, clear melting curves and the highest melting temperature (*T*_m_), indicating maximal biomolecule stability, were determined (Table S4). Subsequently, we identified the optimal concentrations of ECF‐PanT, ECF module, and ECF‐FolT2 along with their respective melting temperatures (Figure S6). The ECF‐FolT2 from *L. delbrueckii* exhibits an average *T*_m_ of 54.6°C, which is higher than that of the *S. pneumoniae* ECF transporter. This difference may be attributed to the fact that *L. delbrueckii* ssp. *bulgaricus* can withstand heat exposure up to 55 °C (Gouesbet et al., 2001). In contrast, the ECF‐PanT and ECF‐module from *S. pneumoniae* show average *T*_m_ values of 39.7 and 39.5°C, respectively. The difference is not statistically significant and although this interpretation requires further validation, the derivative melting temperature graphs for the ECF‐PanT protein (Figure S8) reveal two peaks: a major peak around 39°C that might corresponds to the ECF module and a smaller peak near 60°C possibly representing the S‐component. This hypothesis matches the known melting temperature of the ECF module at approximately 39°C.

Building on this information, we systematically screened a wide range of buffers with various pH values and salt concentrations to investigate their effect on the stability of ECF‐PanT (Figure S7).

Non‐ionic detergent *n*‐dodecyl β‐D‐maltoside 0.05% (DDM) (Rouse et al., 2013) and sodium chloride (50 mM) were added to maintain the solubility of proteins and ionic strength of the buffer; further details are reported in Appendix S1.

**FIGURE 2:**
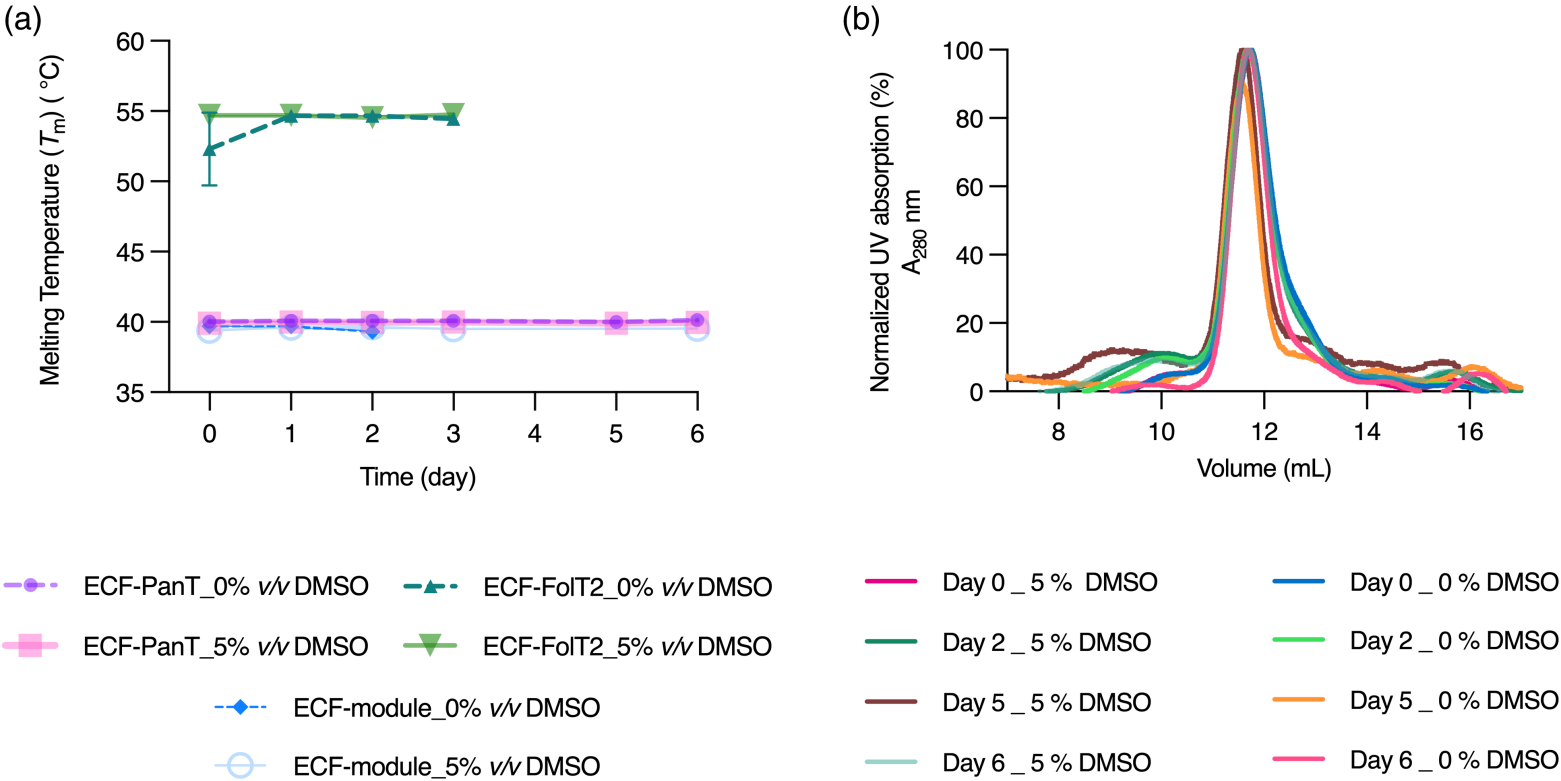
Assessment of ECF‐PanT stability with and without DMSO. (a) Stability of ECF‐PanT, ECF‐FolT2, and ECF‐module was evaluated by monitoring melting temperatures (*T*_m_) at room temperature over 6 days with 0% and 5% (v/v) DMSO. The data represent the mean and standard deviation from two independent experiments. (b) To assess protein stability, ECF‐PanT samples were incubated at room temperature in an optimal buffer (50 mM KPi pH 7.5, 50 mM NaCl, 0.05% DDM) with or without 5% DMSO. UV absorption at 280 nm was measured using size exclusion chromatography (SEC) to determine the consistency of protein absorbance over time. Measurements were performed in technical duplicate to ensure accuracy.

Then, after selecting the KP_i_ buffer due to the highest *T*_m_, we investigated the stability of the ECF‐PanT, ECF‐module and ECF‐FolT2 using TSA for 6 days at room temperature. For ECF‐PanT, we chose the so‐called “optimal buffer” that showed the highest *T*_m_ (50 mM KP_i_ pH 7.5, 50 mM NaCl, 0.05% DDM) (Figure 2), while the ECF‐module and ECF‐FolT2 were incubated in the SEC purification buffer. All the above‐mentioned conditions were tested both in the absence and presence of 5% DMSO (v/v). The addition of DMSO was intended to mimic the SPR conditions used during our compound screening, ensuring consistency throughout the experiments.

Importantly, the melting temperature results, complementary to the corresponding curve shape, indicate that ECF‐FolT2 and the ECF‐module are stable for up to 3 days (Figure 2a), while ECF‐PanT is stable for up to 6 days. In both cases, the buffer contains 5% DMSO (Figures S8–S10). To further validate these results, we loaded the samples on a SEC column followed by SDS–PAGE, where all four bands exhibited no significant degradation (Figures 2b and S11).

### 2.3 Evaluation of known small‐molecule ECF transporter inhibitors

After identifying the optimal buffer and assessing the stability of the ECF transporter proteins, we proceeded to determine the binding affinity (*K*_d_) of small‐molecule inhibitors using SPR. This label‐free technique allows for the investigation of protein–ligand interactions in real time and requires protein immobilization (GE Healthcare Life Science, 2012). Specifically, for the three ECF transporters, we used capture immobilization rather than standard immobilization because it offers several advantages, such as the ordered and uniform orientation of the protein, which does not compromise the protein's binding sites, and the ability to regenerate the surface afterward. The ECF‐PanT, ECF‐module, and ECF‐FolT2, each bearing a histidine tag, were immobilized on a high‐affinity poly‐nitrilotriacetic acid (poly‐NTA) sensor chip, which was initially activated using NiCl_2_. The His‐tagged proteins were then injected over the Ni^2+^‐activated surface to strongly bind to the metal (Knecht et al., 2009). The immobilization resonance unit (RU) levels were 12,000–15,000 for ECF‐PanT, 9000–12,000 for the ECF‐module, and 6000–9000 for ECF‐FolT2.

The SPR assay was used to test in‐house ECF inhibitors **1–6** (Table 1) on the ECF‐PanT, ECF‐module, and ECF‐FolT2 (Bousis et al., 2021; Diamanti et al., 2023; Drost et al., 2022).

**TABLE 1:** Chemical structures of known ECF inhibitors (**1**–**6**) with their IC_50_ values resulted from the *Lactobacillus casei* whole‐cell uptake assay as well as their respective MIC values using *Streptococcus pneumoniae* DSM‐20566 strain.

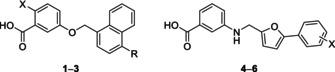				
Compound	X	R	*L. casei* whole‐cell uptake assay IC_50_ (μM)	*S. pneumoniae* DSM‐20566 MIC (μM)
**1**	OH	H	315 ± 15	128
**2**	OH	Br	59 ± 29.5	8
**3**	NHBoc	H	49.2 ± 3.4	16
**4**	*p*‐Cl	‐	321 ± 200^a^	64
**5**	*m*‐Cl	‐	247 ± 78	128
**6**	*o*‐Cl	‐	285 ± 293^a^	128

^a^ The mean IC_50_ values for the compounds was determined from two independent biological replicates. The range of IC_50_ values observed was 6–500 μM, indicating substantial variability in the standard deviation (SD).

As shown in Table 2, compounds **2** and **3** exhibited the lowest dissociation rate constants (*k*_d_) and the highest affinities for both ECF‐PanT and ECF‐FolT2 (Figures S12 and S13). Interestingly, no binding responses were observed for any of the compounds at concentrations up to 200 μM when injected over the ECF module (Figure S14). These data may be consistent with our previous molecular‐dynamics simulations, which predicted compound **1** binding at the interface between the ECF module and the S‐component (Diamanti et al., 2023). The absence of binding to the ECF‐module alone, while showing binding to ECF‐FolT2 and ECF‐PanT, suggests that the S‐component is necessary for compound binding. Moreover, these results can be further explained by considering the conformational changes in the ECF transporter proteins. In fact, the ECF‐module undergoes a conformational change when forming the full ECF complex, which is different from that one of the ECF‐module alone (Thangaratnarajah et al., 2023). On the other hand, the compounds exhibit a consistent binding trend to both ECF‐FolT2 and ECF‐PanT, indicating no substrate dependence. Thus, these findings support the idea that our compounds might act as allosteric inhibitors by binding at the interface between the ECF‐module and the S‐component.

**TABLE 2:** Binding affinities (*K*_D_) and kinetic parameters of compounds **1–6** for the ECF transporter proteins^a^.

ECF transporter	*Streptococcus pneumoniae*					*Lactobacillus delbrueckii*			
	ECF‐PanT				ECF‐module	ECF‐FolT2			
Compound	*k*_a_ (M^−1^ s^−1^)	*k*_d_ (s^−1^)	Kinetic, *K*_D_ (μm)	Equilibrium, *K*_D_ (μm)	*K*_D_ (μm)	*k*_a_ (M^−1^ s^−1^)	*k*_d_ (s^−1^)	Kinetic, *K*_D_ (μm)	Equilibrium, *K*_D_ (μm)
**1**	472 ± 6	0.130 ± 0.001	276 ± 4	277 ± 5	n.a.	620 ± 10	0.181 ± 0.003	294 ± 6	300 ± 20
**2**	384 ± 4	0.0661 ± 0.0004	172 ± 2	170 ± 5	n.a.	411 ± 6	0.0760 ± 0.0007	185 ± 3	190 ± 20
**3**	650 ± 9	0.0643 ± 0.0006	99 ± 1	96 ± 8	n.a.	570 ± 10	0.0755 ± 0.0008	132 ± 3	128 ± 9
**4**	399 ± 4	0.127 ± 0.001	317 ± 3	304 ± 6	n.a.	325 ± 5	0.115 ± 0.001	353 ± 5	340 ± 10
**5**	348 ± 7	0.157 ± 0.002	451 ± 8	420 ± 10	n.a.	570 ± 10	0.187 ± 0.003	328 ± 6	300 ± 30
**6**	376 ± 6	0.147 ± 0.002	391 ± 6	374 ± 6	n.a.	(1 ± 0.1) × 10^5^	22 ± 3	231 ± 5	230 ± 10

Abbreviation: n.a., not applicable due to very weak interaction or no binding.

^a^ *n* = 2.

It is worth mentioning that slightly higher inhibitory activities of the compounds were observed in the whole‐cell assays (Table 1) compared to their binding affinities (Table 2). This variation may be attributed to the dynamic mechanism of action of ECF transporters, in contrast to the relatively ‘locked’ conformation observed in the SPR conditions from the EcfA side, where the His‐tag is attached.

**TABLE 3:** ECF inhibitors **1–6** were tested for toxicity against HepG2 and A549 cells to investigate their effect on cell viability.

Compound	Percent (%) cell viability at 100 μM	
	HepG2 cells	A549 cells
**1**	20 ± 2	111 ± 6
**2**	23 ± 7	124 ± 15
**3**	49 ± 3	86 ± 11
**4**	88 ± 9	80 ± 10
**5**	106 ± 8	102 ± 17
**6**	105 ± 3	103 ± 7

### 2.4 Transport‐activity assay of ECF‐PanT from *S. pneumoniae*

Based on the reported transport activity assay on *L. delbrueckii* (Swier et al., 2016), for the first time, we adapted this assay to a pathogenic bacterium. To do so, we purified the ECF‐PanT transporter, reconstituted it into proteoliposomes, and carried out transport assays with radiolabeled pantothenate. Upon the addition of MgATP, the radiolabeled pantothenate is translocated across the membrane via an ATP‐dependent mechanism. MgATP at 5 mM served as a positive control, supplying the proteins with the energy needed to complete the transport cycle, while MgADP at 5 mM was used as a negative control. The uptake of the radiolabeled substrate into proteoliposomes was measured using a scintillation counter.

**FIGURE 3:**
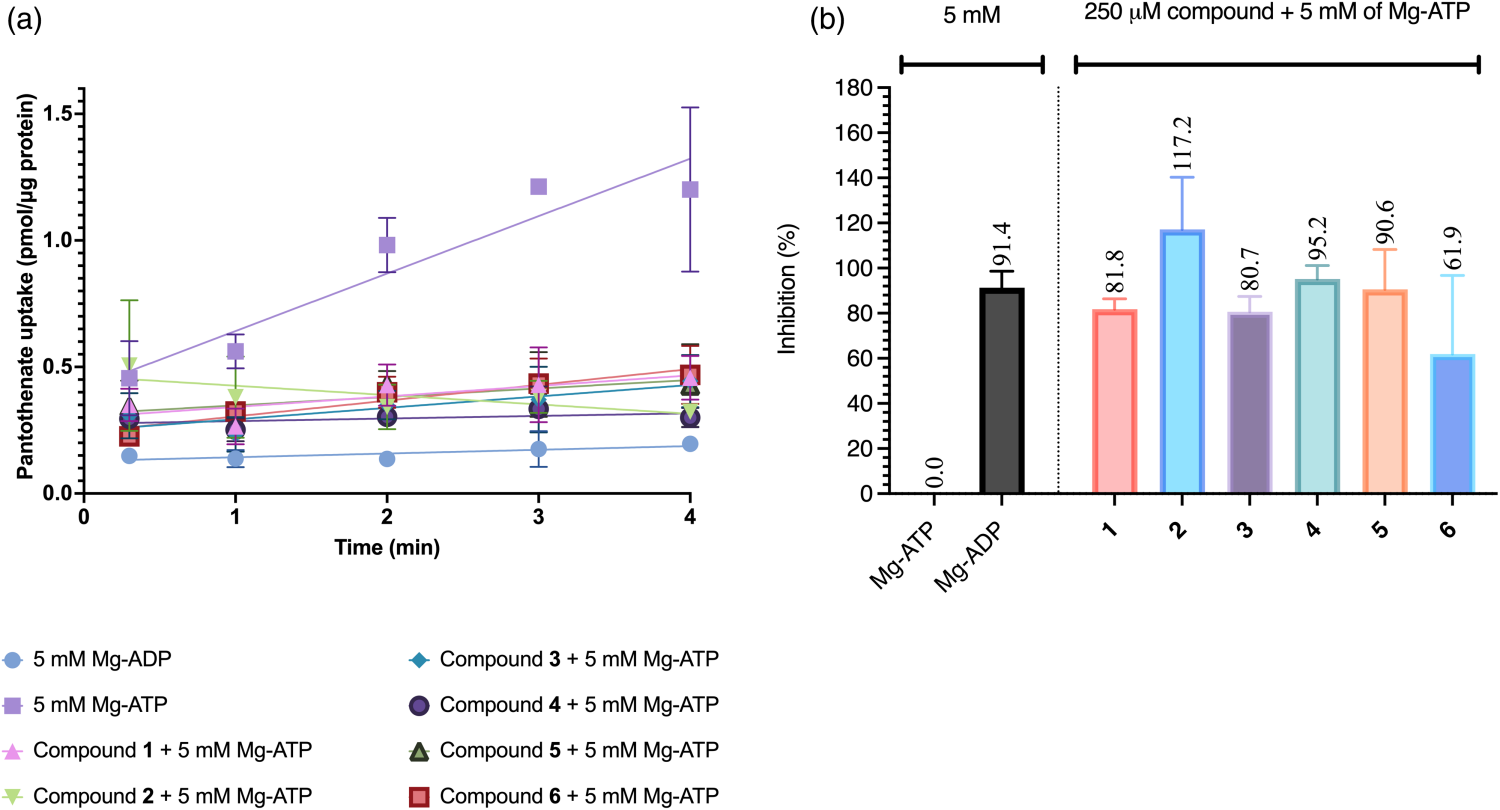
Transport activity assay of reconstituted *S. pneumoniae* ECF‐PanT with a protein‐to‐lipid ratio of 1:125. (a) Time‐course traces of pantothenate uptake over 4 min, showing transport activity in the presence of inhibitors with 5 mM Mg‐ATP. The controls were 5 mM Mg‐ADP (negative control) and 5 mM Mg‐ATP (positive control) alone. The data demonstrate the uptake rates for each condition and was performed in duplicates. (b) Percentage inhibition of pantothenate uptake in presence of ECF inhibitors **1**–**6**, calculated relative to the activity observed with 5 mM Mg‐ATP.

As shown in Figure 3, all compounds (**1**–**6**) exhibited inhibition levels higher than 60% at the tested concentration of 250 μM. Compared to the inhibitory activities observed in the *Lactobacillus casei* whole‐cell uptake assays, which ranged from 38.7% to 95.4% (Bousis et al., 2021; Diamanti et al., 2023; Kiefer et al., 2022), the compounds demonstrated higher inhibition in our transport‐activity assay. The observed disparity in activity may be attributed to several factors, including dissimilarities in the ECF transporter sequences between *L. casei* and *S. pneumoniae*, as well as variations in compound concentrations. However, it is important to note that our study did not provide direct evidence to conclusively link these sequence differences to the observed variations in activity. In addition to sequence variations, differences in assay conditions such as the use of proteoliposome‐based versus whole‐cell uptake assays can also contribute to differences in compound activity and efficacy. Variations in cellular contents and assay conditions, as discussed by Dvorak et al. (2021), can impact the observed results. Further research is needed to explore these potential factors in more detail.

### 2.5 Cytotoxicity evaluation

To investigate the cytotoxic potential of compounds **1**–**6** and to further support the prospective therapeutic use of ECF inhibitors, we evaluated their effect on the viability of human hepatoma (HepG2) and lung cancer (A549) cells. At a concentration of 100 μM, compounds **1** and **2** exhibited substantial cytotoxicity in HepG2 cells (Table 3), reducing viability to 20% and 23%, respectively. In contrast, only minimal effects were observed in A549 cells (viability >100%), suggesting selective toxicity toward liver cells. Also compound **3** displayed moderate cytotoxicity in HepG2 cells (49% viability) and lower toxicity in A549 cells (86% viability). Compound **4** was only slightly toxic in both cell lines, with 88% viability in HepG2 and 80% in A549 cells. Compounds **5** and **6** were non‐toxic against both cell types.

### 2.6 *Galleria mellonella* infection model studies using *S. pneumoniae*

**FIGURE 4:**
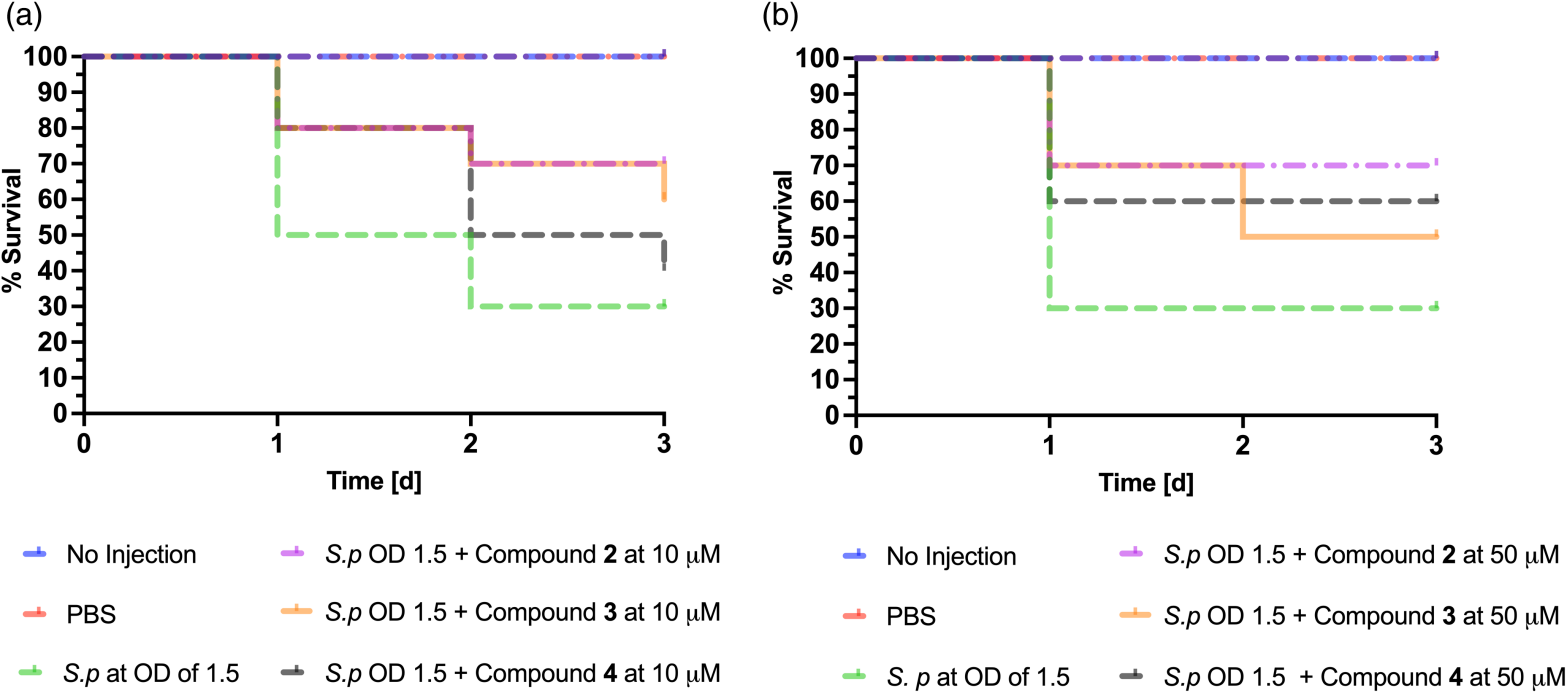
Simple survival analysis (Kaplan–Meier) was performed using GraphPad Prism. (a) The larvae were injected with *S. pneumoniae* (*S.p*) at OD_600_ of 1.5 in the absence and presence of compounds **2**–**4** at 10 μM. The control groups are with no injection and PBS. (b) The larvae were injected with *S. pneumoniae* (*S.p*) at OD_600_ of 1.5 in the absence and presence of compounds **2**–**4** at 50 μM. The control groups are with no injection and PBS.

Next, we examined the antibacterial effect of the ECF inhibitors under in vivo conditions by using *G. mellonella* larvae. This model was adopted based on the work of Alhayek et al. (2022) and serves to assess the efficacy of ECF inhibitors against *S. pneumoniae* induced infection. Larvae were injected with the overnight culture of *S. pneumoniae* DSM‐20566, incubated for 3 days at 37°C and 5% CO_2_ without shaking, and survival rates were monitored daily for 3 days (Figure 4).

Based on the MIC, cytotoxicity, and affinity data, compounds **2**, **3**, and **4** were selected for further investigation. Compound **2** exhibited the strongest antibacterial activity, with a MIC of 8 μM against *S. pneumoniae* and a moderate IC_50_ of 59 μM in the *L. casei* whole‐cell uptake assay. Compound **3** also demonstrated potent antibacterial activity, with a MIC value of 16 μM and good inhibitory activity (IC_50_ of 49.2 μM). While both compounds showed some cytotoxicity in HepG2 cells (in contrast to A549 cells), the dilution effect in vivo would mitigate this potential concern. Compound **4**, despite having a higher MIC of 64 μM, exhibited lower cytotoxicity, making it a safer candidate for further studies. In the *G. mellonella* model, *S. pneumoniae* at an OD_600_ of 1.5 reduced larval survival to 30% after 3 days. The controls (no injection and PBS injection) showed no effect on survival. Treatment with 10 and 50 μM of compound **2** increased survival by up to 70% compared to PBS‐injected larvae, while compound **3** increased the survival to 60% at 10 μM and 50% at 50 μM. Compound **4** improved the survival rate to 40% at 10 μM and 60% at 50 μM. The differences in survival rates between the two concentrations might be attributed to a balance between effective antibacterial activity and cytotoxicity. For compound **2**, the substantial survival rate at 50 μM suggests that its antibacterial effects outweigh potential cytotoxicity. In contrast, the decreased survival of compound **3** at 50 μM indicates that increased cytotoxic effects may limit its efficacy. Notably, compound **4** demonstrated enhanced survival with higher concentrations, indicating its potential as a safer candidate. These results validate our inhibitors as promising candidates for targeting ECF transporters and confirm that the boost in inhibitory activity observed in vitro translates to an improved in vivo effect.

## 3 DISCUSSION

Membrane proteins pose challenges as drug targets, with stability being a crucial factor for experimental success. Using the thermal shift assay (TSA), we demonstrated that the ECF‐module and full complex ECF‐PanT from *S. pneumoniae*, as well as the full complex ECF‐FolT2 from *L. delbrueckii*, are stable over 6 days. This study marks the first report of the isolation, purification, and use of a stable *S. pneumonia* ECF‐module (or EcfTAA′). We further evaluated their stability under assay conditions (room temperature, with and without 5% DMSO) using TSA. This information is essential for drug discovery, serving as a foundation for developing new bioassays.

Notably, the established protocol for membrane proteins can be readily adapted to other ECF membrane proteins, providing vital stability data and assisting in the selection of optimal storage buffers for functional and/or binding assays.

Additionally, we successfully developed a surface plasmon resonance (SPR) protocol to determine the binding affinities of six ligands against three non‐covalently immobilized ECF transporters. This method is particularly effective with His‐tagged target proteins, ensuring uniform binding and orientation on the chip. To our knowledge, we pioneered the use of SPR for assessing ligand binding affinities to transmembrane proteins, identifying HEPES (10 mM HEPES, 50 mM NaCl, 50 μM EDTA, 0.05% (v/v) DDM, pH 7.5) as the optimal buffer for SPR. In this proof‐of‐concept study, we successfully investigated the binding of six small‐molecule inhibitors to three ECF proteins. Furthermore, these conditions can be adapted to a 384‐well plate format, making SPR an accessible and robust screening method for identifying new ECF transporter inhibitors. We also optimized the *G. mellonella* infection model for *S. pneumoniae* to evaluate ECF inhibitors in vivo. These findings pave the way for the exploration and medicinal exploitation of ECF transporters, potentially leading to significant advancements in the treatment of bacterial infections.

## 4 MATERIALS AND METHODS

### 4.1 Transformation, overexpression, and purification

The transformation was carried out using prepared chemically competent *E. coli* cells of strain *MC1061* and modified p2BAD His‐ECF PanT‐StrepII plasmid from *S. pneumoniae NCTC7465*, modified p2BAD His‐ECF from *S. pneumoniae NCTC7465*, and modified p2BAD His‐ECF FolT2 plasmid from *L. delbrueckii* subsp. *bulgaricus ATCC 11842*. The transformed *E. coli* cultures were grown at 37°C overnight on LB‐agar plates with ampicillin, and the cell pellets were stored in 60% glycerol at −80°C. Protein expression and purification followed established protocols (Swier et al., 2016). Supplementary figures corresponding to these methods can be found in Appendix S1 (Figures S3–S5 and Table S3).

### 4.2 Determination of protein stability using TSA

TSA procedure requires incubation of protein and dye in a 96‐well plate (Huynh & Partch, 2015). All the experiments were performed in biological and technical duplicate or triplicate, as stated in the following.

In each step, the end volume was set to 20 μL. The four main components are protein, buffer, and both the GloMelt and ROX reference dye, which were accordingly set to have the desired concentrations. (Table S1 and Figure S6).

The second step is buffer screening for the optimization of protein stability. The composition of each condition in the well plates was set according to Appendix S1 (Figure S7).

The third step was investigating the stability in two different conditions. For this purpose, two conditions were prepared at seven times more than the total volume and incubated at room temperature for almost 1 week, and each day the melting temperature was monitored by running TSA. More detailed procedures can be found in Appendix S1 (Figures S8–S10).

### 4.3 Surface plasmon resonance

In this assay, we used HEPES buffer (10 mM HEPES, 50 mM NaCl, 50 μM EDTA, 0.05% (v/v) DDM, and a pH value of 7.5) as recommended by Xantec Bioanalytics, with a slight modification made to create a more favorable environment for the ECF proteins, enabling their stability to be maintained throughout the assay. Additionally, the protein was maintained in the buffer containing 5% (v/v) DMSO. For a more in‐depth description of the procedures, please refer to Appendix S1.

### 4.4 Transport‐activity assay

The assay was performed based on the published protocol (Swier et al., 2016). The only modification was reconstitution of *S. pneumoniae* ECF‐PanT in a 1:125 (w/w) ratio of protein to *E. coli* polar lipids into large unilamellar vesicles and using radiolabeled pantothenate for uptake.

### 4.5 HepG2 in vitro MTT assay

The assay was performed based on the published protocol (Haupenthal et al., 2007), with some modifications, which can be found in Appendix S1.

#### 4.5.1 G. mellonella *infection model*

*G. mellonella* larvae were used as an in vivo infection model to test selected ECF inhibitors, following the methodology established by Alhayek et al. (2022). *S. pneumoniae* DSM‐20566 was cultured in Todd–Hewitt medium with 0.1% choline at 37°C with 5% CO_2_ until an OD600 of 1.5 or higher was reached. Larvae were infected by injecting 10 μL of the culture into the left proleg and incubated at 37°C, 5% CO_2_ for 72–96 h. Survival was monitored daily. Control groups received either PBS or no injection. Compounds **2**–**4** significantly improved larvae survival compared to the untreated control. *The* larvae used in this study were purchased from VALOMOLIA Company (Strasbourg, France).

## AUTHOR CONTRIBUTIONS

**Atanaz Shams:** Writing – original draft; conceptualization; methodology; formal analysis; validation; investigation; writing – review and editing; visualization; supervision. **Spyridon Bousis:** Conceptualization; writing – review and editing; methodology; supervision. **Eleonora Diamanti:** Conceptualization; writing – review and editing; methodology; supervision. **Walid A. M. Elgaher:** Writing – review and editing; formal analysis; supervision; methodology; validation; visualization. **Lucie Zeimetz:** Formal analysis; investigation. **Jörg Haupenthal:** Supervision; writing – review and editing; validation. **Dirk J. Slotboom:** Conceptualization; supervision; writing – review and editing; validation. **Anna K. H. Hirsch:** Project administration; resources; funding acquisition; supervision; conceptualization; writing – review and editing.

## CONFLICT OF INTEREST STATEMENT

The authors declare no conflicts of interest.

## Supporting information


**APPENDIX S1:** The file “SM_Expression and characterization of ECF transporters_Shams” describes the below mentioned methods in more detail.

## Data Availability

The data that support the findings of this study are available from the corresponding author upon reasonable request.
