# Supplementary material for: Expression and characterization of pantothenate energy‐coupling factor transporters as an anti‐infective drug target
Source: Protein Sci. 2024 Oct 29;33(11):e5195. doi: 10.1002/pro.5195 (PMC11521937; doi:10.1002/pro.5195)
Supplement: Supplementary file 1 — APPENDIX S1: The file “SM_Expression and characterization of ECF transporters_Shams” describes the below mentioned methods in more detail. [file PRO-33-e5195-s001.pdf]

**Expression and characterization of pantothenate energy-coupling factor (ECF) transporters as an anti-infective drug target**

*Atanaz Shams*[1,2], *Spyridon Bousis*[1,2,3], *Eleonora Diamanti*[4,1], *Walid A. M. Elgaher*[1], *Lucie Zeimetz*[1,2], *Jörg Haupenthal*[1], *Dirk. J. Slotboom*[5,2], *Anna K. H. Hirsch*[1,2*]

[1] Helmholtz Institute for Pharmaceutical Research Saarland (HIPS) – Helmholtz Centre for Infection Research (HZI), Department of Drug Design and Optimization, 66123, Saarbrücken, Germany

[2] Saarland University, Department of Pharmacy, Campus Building E8.1, 66123, Saarbrücken, Germany

[3] Stratingh Institute for Chemistry and Technology, Faculty of Science and Engineering, University of Groningen, Nijenborgh 4, 9747AG, Groningen, The Netherlands

[4] Department of Pharmacy and Biotechnology, Alma mater studiorum - Università di Bologna - Via Zamboni, 33 - 40126 Bologna

[5] Groningen Biomolecular Sciences and Biotechnology Institute, University of Groningen, Nijenborgh 4, 9747AG, Groningen, The Netherlands

*Corresponding author: Anna K. H. Hirsch, Helmholtz-Institut für Pharmazeutische Forschung Saarland (HIPS), Campus E8.1, 66123 Saarbrücken, +4968198806-2100, anna.hirsch@helmholtz-hips.de.

**- Supplementary Material -**

Table of Contents

*Sequence alignment* ........ 3
*Building of S. pneumonaie ECF-PanT homology model* ........ 7
*Transformation, overexpression, and purification* ........ 8
*Stability determination* ........ 10
**Optimal concentrations for protein and dye** ........ 10
***S. pneumoniae* ECF-PanT buffer screening** ........ 11
**Protein thermal stability: 6-Day study** ........ 13
*Evaluation of stability data resulted from TSA* ........ 15
*Protein–ligand interaction study using TSA* ........ 15
*Surface plasmon resonance (SPR)* ........ 16
*In vitro cytotoxicity evaluation* ........ 19

## Sequence alignment

The NCBI BLAST program was utilized to conduct the BLAST analysis with specific parameters, including Blastp and an E-value threshold of 0.05 and scoring matrix of BLOSUM62 (Altschul et al., 1997, 2005). The subject protein under examination was *S. pneumoniae* ECF-PanT while the query proteins were *L. delbrueckii* ECF-FolT2 (Swier et al., 2016), *L. delbrueckii* ECF-PanT (Setyawati et al., 2020), and *Levilactobacillus brevis* ECF-PanT (Zhang et al., 2014) (Table S1).

**Table S1: List of proteins with their respective source organisms, Protein Data Bank (PDB) identifiers, and UniProtKB accession numbers.**

| Protein name | PDB ID | UniProtKB ID EcfS | UniProtKB ID EcfT | UniProtKB ID EcfA1 | UniProtKB ID EcfA2 |
|---|---|---|---|---|---|
| *S. pneumoniae* ECF-PanT | n.a.[a] | A0A064C5C4 | A0A4L7ULF4 | Q04HV7 | Q97N51 |
| *L. delbrueckii* ECF-FolT2 | 5JSZ | Q1G929 | A0A061BSU4 | Q1GBJ0 | Q1GBI9 |
| *L. delbrueckii* ECF-PanT | 6ZG3 | Q1GBG0 | Q1GBI8 | Q1GBJ0 | Q1GBI9 |
| *Levilactobacillus. brevis* ECF-PanT | 4RFS | Q03SM0 | Q03PY7 | Q03PY5 | Q03PY6 |

[a] not applicable.

Table S2 provides an overview of the BLAST results, suggesting moderate sequence similarity between the subject protein, *S. pneumoniae* ECF-PanT, and the query proteins. *L. delbrueckii* ECF-PanT appears to exhibit the closest match, with a Max Score of 776, 99% query coverage, and 40.80% identity, implying a relatively strong alignment. Similarly, *L. delbrueckii* ECF-FolT2 shows a Max Score of 745, 95% coverage, and 40.61% identity, indicating a comparable degree of similarity. *L. brevis* ECF-PanT, on the other hand, displays a lower Max Score of 341 but a significantly higher Total Score of 1308, with 95% coverage and 36.36% identity, which might point to a slightly weaker sequence similarity, though the alignment still spans a substantial portion of the sequence. These results suggest a varying degree of conservation across the proteins, but further analysis would be required to confirm their functional relationships.

**Table S2: Summary of BLAST search results for the protein sequences analyzed in this study. The table includes the maximum and total scores, query coverage (%), percent identity (%), E-values, and accession length. These metrics provide insights into the similarity and alignment quality between the query proteins and the target sequences.**

| description | Max Score | Total Score | Query Cover | E Value | Per. Ident | Acc. Len | Accession |
|---|---|---|---|---|---|---|---|
| *L. delbrueckii* ECF-PanT | 776 | 776 | 99% | 0.0 | 40.80% | 1041 | Query_7093401 |
| *L. delbrueckii* ECF-FolT2 | 745 | 745 | 95% | 0.0 | 40.61% | 1010 | Query_7093402 |
| *L. brevis* ECF-PanT | 341 | 1308 | 95% | 1e-104 | 36.36% | 1038 | Query_7093403 |

In this study, the multiple sequence alignment with all the query and subject proteins was produced by T-Coffee (Di Tommaso et al., 2011; Notredame, Higgins, & Heringa, 2000), and ESPript 3.0 was employed to improve the visual representation of the multiple sequence alignments, enabling clearer depiction of sequence similarities and conservation patterns. For consistency with the BLAST analysis, the BLOSUM62 substitution matrix was selected as the scoring system for visualizing sequence similarity (Figure S1). (Robert & Gouet et al., 2014.)

```
                1        10        20        30                 40          50
S.p_ECF-PANT    MKKRSNIAPIAIFFATMLVIHFLSSLIFNLFPFPIKPT........IVHIP.VIIASIIY
L.d_ECF-PANT    M...........................................................
L.d_ECF-FOLT2   MKSESKVSSKLELRE.LVLLAMVIAIKVILGQFKVGNA........TLQVGLGFIGSVML
L.b_ECF-PANT    MTENI.I.S................VDHLTYQYDENQAPALTDVSFTVHAG.EWLAIVGH

                        60        70        80        90       100       110
S.p_ECF-PANT    GPRVGVTLG.FLMGLLSLTVNTITILPTSYLFSPFVPNGNIYSAIIAIVPRILIGLTPYL
L.d_ECF-PANT    ............................................................
L.d_ECF-FOLT2   GYLFGPWWG.FAGGALSDLVSSVIFGNLGGFFIGFTL....TAALGPMIYGFFLYKQPIQ
L.b_ECF-PANT    NGSGKSTLAKSLDGLLPFTQGSVTVGG..........................ITLTPET

                       120       130       140       150       160       170
S.p_ECF-PANT    VYKLMKNKTGLILAGALGSLTNTIFVLGGIFFLFGNVYNGNIQLLLATVISTNSIAELVI
L.d_ECF-PANT    ............................................................
L.d_ECF-FOLT2   IWRVIASVICVTVICNIGLNTLWVSMMYGINFMV..........AL.............S
L.b_ECF-PANT    VWQVREQ.IGMIFQNPDNQ...FVGATVEDDVAF..........GL............EN

                       180                         190       200       210
S.p_ECF-PANT    SAILTLAIVPRLQTL................KKMDSMILGRYIPGDSIVHRLDPRSKLLA
L.d_ECF-PANT    ..................................SKIIIGRYLPGTTFVYRVDPRAKLLT
L.d_ECF-FOLT2   SRILKEMITPWIQMVAVWFILEGLSRVKLSRKFMSKIIIGRYLPGTTFVYRVDPRAKLLT
L.b_ECF-PANT    RQISRDEMVPRVQ.........................AALAQVGMTSFAQREPS.....

                    220       230       240       250       260       270
S.p_ECF-PANT    MMLLILIVFWANNPLTNLILFIATGIFIALSGVSLSFFIQGLKSIFFLIAFTTIFQLFFI
L.d_ECF-PANT    TFYFIIMIFLANNWVSYLVISIFGLAYVFATGLKARVFWDGVKPMIWMIVFTSLLQTFFM
L.d_ECF-FOLT2   TFYFIIMIFLANNWVSYLVISIFGLAYVFATGLKARVFWDGVKPMIWMIVFTSLLQTFFM
L.b_ECF-PANT    ...........................SLSGGQKQRVALAGI..................

                    280       290       300       310       320       330
S.p_ECF-PANT    SNGNVLFEFSFVRITDYALQQAGIIFCRFVLIIFFSTLLTLTTMPLSLASAVEALLAPLK
L.d_ECF-PANT    AGGKVYWHWWIFTLSSEGLINGLYVFIRFAMIILVSTVMTVTTKPLEIADAMEWMLTPLK
L.d_ECF-FOLT2   AGGKVYWHWWIFTLSSEGLINGLYVFIRFAMIILVSTVMTVTTKPLEIADAMEWMLTPLK
L.b_ECF-PANT    ......................................VAIAPK.ILILDEATSMLDPQG

                    340       350       360        370       380
S.p_ECF-PANT    RVKVPVHEIGLMLSMSLRFVPTLMDDTTRIMNAQ.KARGVDFGEGSIVQKVKAM.....I
L.d_ECF-PANT    LFKVNVGMISLVISIALRFVPTLFDQTVKIMNAQ.RSRGADFNDGGLVKRAKSV.....V
L.d_ECF-FOLT2   LFKVNVGMISLVISIALRFVPTLFDQTVKIMNAQ.RSRGADFNDGGLVKRAKSV.....V
L.b_ECF-PANT    R....IEMLAIVRQLRQQQNLTVISITHDIDEAASADRVLVIDDGRLVDEAVPSQIFERG

              390       400       410         420        430       440
S.p_ECF-PANT    PILIPLFATSLKRADSLAIAMEARGYQGG.KGRSQYRQLKWTLKDTLTILVILVLGCC.L
L.d_ECF-PANT    PMLVPLFIDSLEVALDLSTAMESRGYKGS.EGRTRYRILEWSKVDLIPVAYCLLLTILMI
L.d_ECF-FOLT2   PMLVPLFIDSLEVALDLSTAMESRGYKGS.EGRTRYRILEWSKVDLIPVAYCLLLTILMI
L.b_ECF-PANT    TQLVEMG.LDLPFTEKLKAALRQRGITPPTTYQTAAEMEEWLWQS...............

                  450       460       470       480       490       500
S.p_ECF-PANT    FFLKSMGIALENVNFTYQEGTPLASAALSDVSLTIEDGSYTALIGHTGSGKSTILQLLNG
L.d_ECF-PANT    TTRKHMAIKFENVSYVYSPGSPLEAIGLDQLNFSLEEGKFIALVGHTGSGKSTLMQHFNA
L.d_ECF-FOLT2   TTRKHMAIKFENVSYVYSPGSPLEAIGLDQLNFSLEEGKFIALVGHTGSGKSTLMQHFNA
L.b_ECF-PANT    .LSNTMAIAFEHVTYTYQAGTPMAHTALTDVSLTVPDRGYLAIIGHTGSGKSTLIQQLNA

                  510       520       530       540       550       560
S.p_ECF-PANT    LLVPSQGSVRVFDTLITSTSKNKDIRQIRKQVGLVFQFAENQIFEETVLKDVAFGPQNFG
L.d_ECF-PANT    LLKPTSGKIEIAGYTITPETGNKGLKDLRRKVSLAFQFSEAQLFENTVLKDVEYGPRNFG
L.d_ECF-FOLT2   LLKPTSGKIEIAGYTITPETGNKGLKDLRRKVSLAFQFSEAQLFENTVLKDVEYGPRNFG
L.b_ECF-PANT    LLKPTSGTIKIDEFTITPETTNAALKPLRQHVGMVFQFPENQLFEETVRQDIAFGPKNFG

                  570       580       590       600       610       620
S.p_ECF-PANT    VSEEDAVKTAREKLALVGIDESLFDRSPFELSGGQMRRVAIAGILAMEPAILVLDEPTAG
L.d_ECF-PANT    FSEDEAREAALKWLKKVGLKDDLIEHSPFDLSGGQMRRVALAGVLAYEPEIICLDEPAAG
L.d_ECF-FOLT2   FSEDEAREAALKWLKKVGLKDDLIEHSPFDLSGGQMRRVALAGVLAYEPEIICLDEPAAG
L.b_ECF-PANT    MADADALALADEMLTTVGLDQSYAERSPFELSGGQMRRVAIAGVLAMQPKVLVLDEPTAG

                  630       640       650       660       670       680
S.p_ECF-PANT    LDPLGRKELMTLFKKLH.QSGMTIVLVTHLMDDVAEYANQVYVMEKGRLVKGGKPSDVFQ
L.d_ECF-PANT    LDPMGRLEMMQLFKDYQ.AAGHTVILVTHNMDDVADYADDVLALEHGRLIKHASPKEVFK
L.d_ECF-FOLT2   LDPMGRLEMMQLFKDYQ.AAGHTVILVTHNMDDVADYADDVLALEHGRLIKHASPKEVFK
L.b_ECF-PANT    LDPQGRQEMMRLFARLHQEQGLTIVLVTHQMEDVAQYAEQVAVMHEGRLMKFGTPADVFS
```

```
                   690       700       710       720             730
S.p_ECF-PANT    DVVFMEEVQLGVPKITAFCKRLADRGVSFKRLPIKIEEFKESL......NGMK.....SI
L.d_ECF-PANT    DSEWLQKHHLAEPRSARFAAKLEAAGLKLPGQPLTMPELADAIKQ..SLKGGEHEMSDNI
L.d_ECF-FOLT2   DSEWLQKHHLAEPRSARFAAKLEAAGLKLPGQPLTMPELADAIKQ..SLKGGEHEMSDNI
L.b_ECF-PANT    NREWLQDHQLDVPQAAQFARRLRDRGLTFPKQPLTADQLADYLAQQWAQRGADHVMSNFI

                        740       750       760       770       780       790
S.p_ECF-PANT    ID....VKNLSFRYKENQNYYDVKDITFHVKRGEWLSIVGHNGSGKSTTVRLIDGLLEAE
L.d_ECF-PANT    IS....FDHVTFTYPDSPR.PALSDLSFAIERGSWTALIGHNGSGKSTVSKLINGLLAPD
L.d_ECF-FOLT2   IS....FDHVTFTYPDSPR.PALSDLSFAIERGSWTALIGHNGSGKSTVSKLINGLLAPD
L.b_ECF-PANT    FGRYLPLDSVV..HRLDPR.AKLM.LSFCY..............................

                           800       810       820       830       840
S.p_ECF-PANT    ...SGEIVIDGQRLTEENVWNIRRQIGMVFQNPDNQFVGATVEDDVAFGLENQGLSRQEM
L.d_ECF-PANT    DLDKSSITVDGVKLGADTVWEVREKVGIVFQNPDNQFVGATVSDDVAFGLENRAVPRPEM
L.d_ECF-FOLT2   DLDKSSITVDGVKLGADTVWEVREKVGIVFQNPDNQFVGATVSDDVAFGLENRAVPRPEM
L.b_ECF-PANT    .........IIVVFLANNIWSYAILIAF........TVGAILSSKISLGFFLKGI.RPLL

                 850       860
S.p_ECF-PANT    KKRVEEALALVGMLDF........................................KKR.
L.d_ECF-PANT    LKIVAQAVADVGMADY........................................ADS.
L.d_ECF-FOLT2   LKIVAQAVADVGMADY........................................ADS.
L.b_ECF-PANT    WLIVFTVVLQLLFSPAGGHTYFHWAFINVTQDGLINAGYIFVRFLLIIMMSTLLTLSTQP

                             870       880       890       900       910
S.p_ECF-PANT    ...........EPARLSGGQKQRVAIAGVVALRPAILILDEATSMLDPEGRRELIGTVKG
L.d_ECF-PANT    ...........EPSNLSGGQKQRVAIAGILAVKPQVIILDESTSMLDPEGKEQILDLVRK
L.d_ECF-FOLT2   ...........EPSNLSGGQKQRVAIAGILAVKPQVIILDESTSMLDPEGKEQILDLVRK
L.b_ECF-PANT    LDIATGLASLMKPLRWVKVPVDTLAMMLSIALRFVPTLMDEATKIMNAQRARGVDFGEGG

S.p_ECF-PANT    ............................................................
L.d_ECF-PANT    ............................................................
L.d_ECF-FOLT2   ............................................................
L.b_ECF-PANT    LFKQAKSLIPLMVPLFMSAFNRAEDLSTAMEARGYQDSEHRSQYRILTWQRRDTVTWLLF

                                                               920       930
S.p_ECF-PANT    .....................IR.......................KDYDMTVISITHDL
L.d_ECF-PANT    .....................IK.......................EDNNLTVISITHDL
L.d_ECF-FOLT2   .....................IK.......................EDNNLTVISITHDL
L.b_ECF-PANT    LLGFVAILIFRHWMTRHKTFRLVVDALLMAIVLLQNLVPFLGYIPFGPFSMTLIGLTVIV

S.p_ECF-PANT    EEVAM.......................................................
L.d_ECF-PANT    EEAAG.......................................................
L.d_ECF-FOLT2   EEAAG.......................................................
L.b_ECF-PANT    AGSALGPRDGLLIGGFWGLITFVRAFTWPSSPVAPLIFTNPLISILPRLLMGLVAGSLYL

                                       940       950       960       970
S.p_ECF-PANT    ......................SDRVLVMKKGEIESTSSPRELFSRND.LDQIGLDDPFA
L.d_ECF-PANT    ......................ADQVLVLDDGQLLDQGKPEEIFPKVEMLKRIGLDIPFV
L.d_ECF-FOLT2   ......................ADQVLVLDDGQLLDQGKPEEIFPKVEMLKRIGLDIPFV
L.b_ECF-PANT    WGRHRQWSMRQAMQVAAGCAALTNTVLVLGLVFLF........................Y

                                      980       990                 1000
S.p_ECF-PANT    ..................NQLKKSLSQNGYDLPENYLTESELE........DKLWELL..
L.d_ECF-PANT    ..................YRLKQLLKERGIVLPDEIDDDEKLV........QSLWQLN..
L.d_ECF-FOLT2   ..................YRLKQLLKERGIVLPDEIDDDEKLV........QSLWQLN..
L.b_ECF-PANT    QTPAVATAFGATGNQTLGYVLMISLFTNGI..PELILD..VLVAPLIAMPLRRQWERLKP

S.p_ECF-PANT    .....
L.d_ECF-PANT    ..SKM
L.d_ECF-FOLT2   ..SKM
L.b_ECF-PANT    QTTK.
```

**Figure S1.** Visualization of multiple sequence alignment (MSA) performed by T-Coffee, visualized using ESPript online tool.

## Building of *S. pneumonaie* ECF-PanT homology model

The ECF-PanT homology model was generated using Molecular Operating Environment (MOE), version 2022.02, Chemical Computing Group ULC, 910–1010 Sherbrooke St. W. Montreal, Quebec, H3A 2R7, Canada.

The sequences of *L. delbrueckii* ECF-FolT2 (PDB ID: 5JSZ), and the AlphaFold-predicted *S. pneumoniae* ECF-A (AF-Q04HV7-F1-v4), ECF-A′ (AF-Q97N51-F1-v4), ECF-T (AF-A0A4L7ULF4-F1-v4), and PanT S-component (AF-A0A064C5C4-F1-v4) were imported into the sequence editor window, and each subunit of the *S. pneumoniae* ECF-PanT transporter was allocated to the corresponding chain of the *L. delbrueckii* ECF-FolT2. From protein panel, homology model option was selected and the sequence of *S. pneumoniae* ECF-PanT was indicated as source, and the *L delbrueckii* ECF-FolT2 as template with align sequence to template option was chosen. Model scoring was set to RMSD to mean, and refinement gradient limit to 0.5.

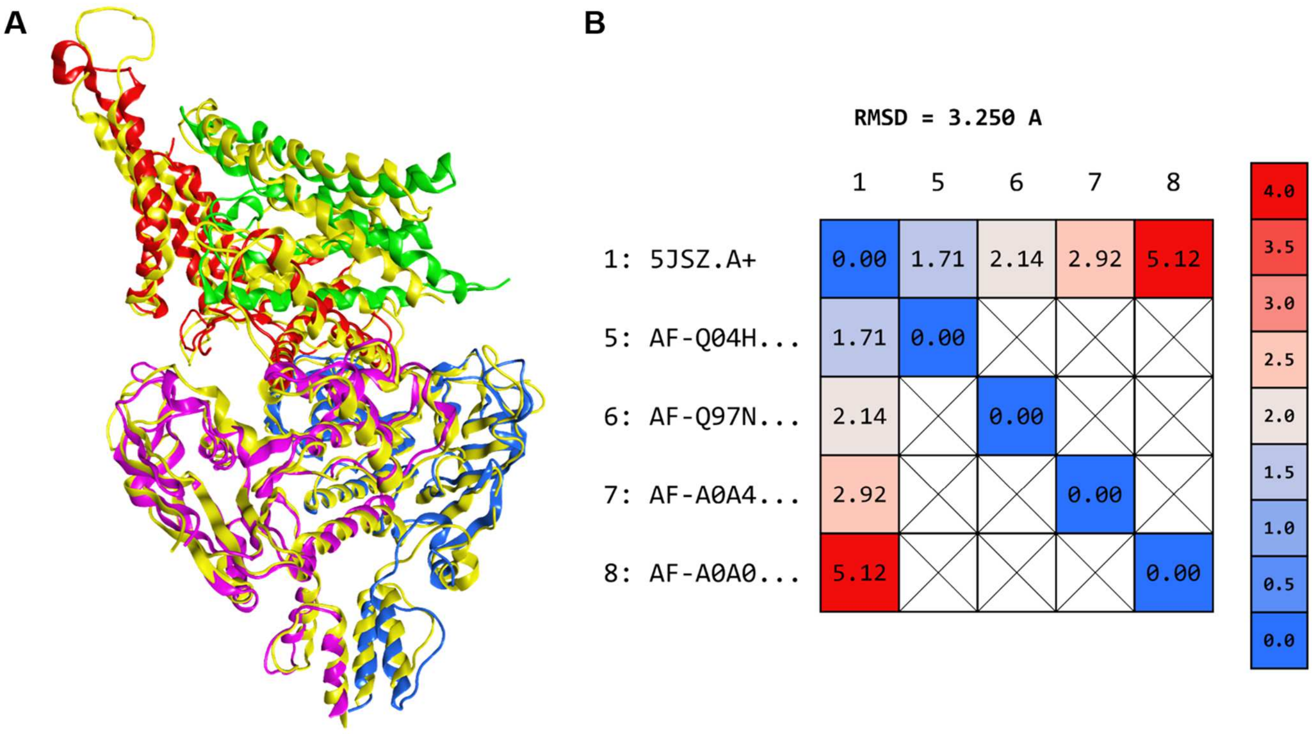


**Figure S2.** (A) Alignment of *L. delbrueckii* ECF-FolT2 structure (yellow) (PDB ID: 5JSZ) and *S. pneumonaie* ECF-PanT homology model: ECF-A (blue), ECF-A′ (magenta), ECF-T (red), and PanT S-component (green). (B) RMSD values of the whole homology structure and individual subunits.

## Transformation, overexpression, and purification

The transformation, expression and purification were performed according to established procedures *(Swier et al., 2016)*

For confirmation of protein presence and effectiveness of purification, SDS-PAGE was carried out (Figure S3–S5). The molecular weight of each component of ECF-PanT, ECF-module, and ECF-FolT2 was computed using ExPASY (Table S3).

**Table S3. The molecular weight (MW) of ECF-PanT, ECF-module from *S. pneumoniae*, and ECF-FolT2 from *L. delbrueckii*.**

| Protein | *S. pneumoniae* ECF-PanT (MW in KDa) | *S. pneumoniae* ECF-module (MW in KDa) | *L. delbrueckii* ECF-FolT2 (MW in KDa) |
|---|---|---|---|
| EcfT | 29,47758 | 29,47758 | 30,26036 |
| EcfA | 32,53077 | 32,53077 | 31,63017 |
| EcfA′ | 30,55329 | 30,55329 | 30,84509 |
| EcfS | 21,31286 | - | 19,42754 |
| Total | 113,8745 | 92,56164 | 112,16316 |

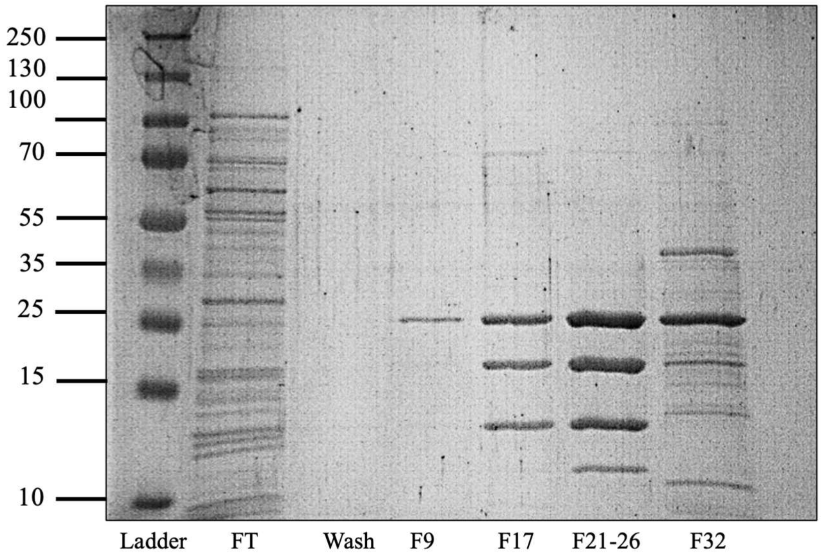


**Figure S3**. The 12% SDS-PAGE from purified ECF-PanT. FT represents the flow through the sample, the wash is the sample collected after wash, and F9, F17, F21-26, and F32 represent the elution fractions, from which A21-26 is considered the purified protein with separation of 4 different components of energy-coupling factor transporter.

Figure S4 shows SDS-PAGE for ECF-FolT2. This time additionally we investigated the effect of temperature as well as freezing and thawing on the protein. For this purpose, we had two conditions, in which the protein was incubated at 95 °C and room temperature (RT) to investigate the purity of the protein after purification. The two ATP-binding protein bands are observable at 95 ºC and after one thaw, but EcfT and EcfS (FolT2) bands are not present at 95 ºC, which can probably be due to protein precipitation at 95 °C, although a high concentration of SDS is present. As a result, heating the mixture should be avoided.

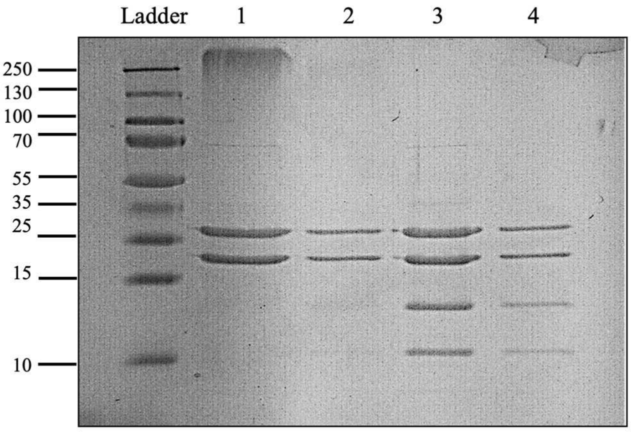


**Figure S4.** 12% SDS-PAGE gel from Purified ECF-FolT2 fractions. 1) incubated at 95 ºC for 5min 2) after one-time thaw incubated at 95 ºC for 5 min 3) incubated at RT for 5min 4) after one-time thaw incubated at RT for 5min.

In the following (Figure S5), 12% SDS-PAGE was performed to confirm the purity of the ECF-module. Indeed, the ECF-module contains the three domains EcfT, EcfA, and EcfA′.

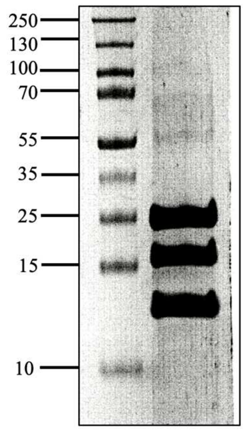


**Figure S5**. 12% SDS-PAGE gel from Purified ECF-module resulting from SEC.

SDS-PAGE results for ECF transporter proteins show four bands that do not align with their expected molecular weights. It could be due to protein conformational changes which means that the structural changes or conformational flexibility in proteins can influence their electrophoretic mobility. If the ECF transporter proteins due to release from their native environment undergo conformational changes that affect their shape or charge distribution, it can lead to different migration patterns on SDS-PAGE.

## Stability determination

### Optimal concentrations for protein and dye

TSA was used to determine the optimal concentrations of GloMelt dye and the ECF-PanT protein. To do so, we monitored how the melting temperature and shape of the curve changed as a function of different concentrations of ECF-PanT protein and GloMelt dye. Subsequently, by adding various final concentrations of protein, starting from 0.73 mg/mL which was the highest concentration of protein after purification, to 0.05 mg/mL (Table S2), as well as different concentrations of GloMelt (2x, 1x, and 0.5x). The effect of ROX 0.5 µM was investigated by addition to different concentrations of GloMelt; the total volume was set to 20 µL as well but the volume for each component was altered to keep the desired concentration. Then, the well plate was centrifuged, and the melting temperature was measured using a StepOnePlus® instrument. In run-setup, the start- and end-temperature were set to 20 °C and 95 °C, respectively; the heating rate was set to 0.5 °C per minute. The melting temperatures were analyzed using protein thermal shift software.

The lowest concentration of dye 0.5x and the protein 0.05 mg/mL were not enough to generate a good signal (Table S4). The experiment was carried out in two biological replicates for biological variability and two technical replicates for experimental precision.

**Table S4. Optimization of dye and the ECF-PanT protein concentration.** Orange-color highlight exhibits inadequate concentrations either due to low value or unclear curve shape.

| ECF-PanT (mg/mL) | GloMelt | $T_m$ (°C) |
|---|---|---|
| 0.73 | 2 x | 39.53 ± 0 |
| | 1 x | 39.45 ± 0.07 |
| | 0.5 x | 39.55 ± 0.02 |
| 0.20 | 2 x | 39.68 ± 0.14 |
| | 1 x | 40.04 ± 0.07 |
| | 0.5 x | 40.43 ± 0 |
| 0.10 | 2 x | 39.83 ± 0 |
| | 1 x | 40.49 ± 0.07 |
| | 0.5 x | 41.18 ± 0.14 |
| 0.05 | 2 x | Unknown |
| | 1 x | Unknown |
| | 0.5 x | Unknown |

As a result, the optimal concentrations of GloMelt and ROX dyes were the same as the recommended amount for real-time PCR Instrument x1 and 0.5 µM (4). In the next step, consequently, the concentration of dyes was kept constant, while the protein concentrations for ECF-PanT, ECF-module, and ECF-FolT2 proteins were set to be between approximately 0.2 and 1 mg/mL. In Figure S6, a summary of the melting temperatures of all three proteins is shown. With this information, we can conclude the control melting temperature and start to screen different buffers and their effect on protein $T_m$ and finally investigate the stability.

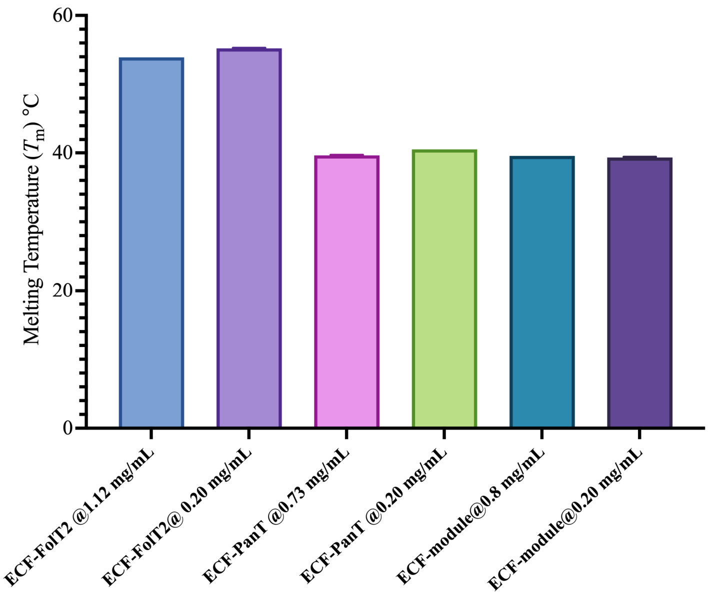


**Figure S6.** The melting temperature ($T_m$) of the targeted proteins with constant GloMelt and ROX dye at x1 and 0.5 µM and different concentrations of proteins. The highest concentration is 10 µL of each protein stock from purification in the final volume of 20 µL, and the lowest concentration is approximately a quarter of the high value.

## *S. pneumoniae* ECF-PanT buffer screening

For buffer screening (Figure S7), four different buffers were chosen: 50 mM $KP_i$, 0.5 M MES, 20 mM HEPES, and 50 mM Tris buffer. For each buffer, three different pH values, and for each pH, three different salt concentrations were selected. In addition, 0.05% DDM was added to each buffer. The optimal protein concentration of 0.5 mg/mL was used for this step. The ROX and GloMelt dye concentrations were 0.5 µM and 1x, respectively. The total volume was set to 20 µL with a similar setup as explained above

As a negative control, no protein was added which shows no melting temperature and wasn't included in the graphs. The positive control was the size exclusion chromatography (SEC) purification buffer *e.g.,* 50 mM $KP_i$ buffer, pH 7.5 with 150 mM NaCl, and 0.05 % DDM which also is used for storage and most of the experiments and is shown in bold format.

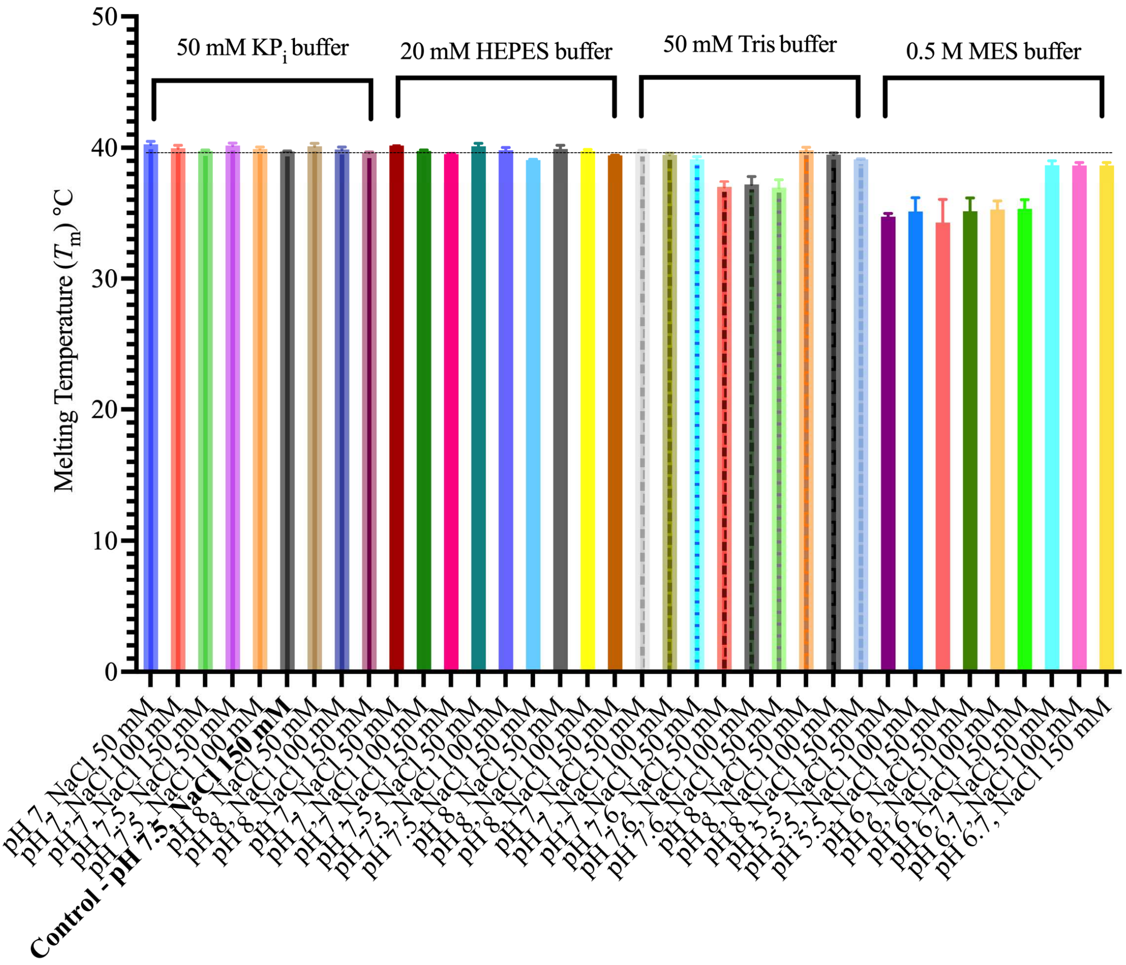


**Figure S7.** Buffer screening for *S. pneumoniae* ECF-PanT, using four different buffers with three different pH and salt concentrations for each. The control is 50 mM $KP_i$ buffer, pH 7.5 with 150 mM NaCl and 0.05 % DDM.

Among all the conditions tested (Figure S7), potassium phosphate (KPi), as well as HEPES buffers show approximately the same melting temperature (i.e., Tm of 39.7 ºC) as the control buffer e.g. size exclusion chromatography (SEC) buffer (e.g., 50 mM KPi buffer, pH 7.5 with 150 mM NaCl, and 0.05 % DDM). Tris buffer with a pH value of 7.6 and MES buffer at all tested pH values (especially pH 5.5 and 6), showed lower melting temperatures for ECF-PanT (i.e., average Tm of 35.4 ºC). Based on these results, it was determined that $KP_i$ and HEPES buffers generally exhibited higher melting temperatures compared to the control buffer used for SEC purification and storage.

**Protein thermal stability: 6-Day study**

Based on this observation, we proceeded to investigate the stability of all three proteins over a period of 6 days, with the proteins incubated in the selected buffer. For the ECF-PanT protein, we selected the optimal buffer based on the results obtained from the buffer screening process, which exhibited a higher melting temperature (Tm). This buffer was denoted as the "optimal buffer" and comprised, for instance, 50 mM $KP_i$ at pH 7.5, 50 mM NaCl, and 0.05% DDM. Conversely, the ECF-module and ECF-FolT2 proteins were incubated in the buffer employed during the size exclusion chromatography (SEC) purification, namely 50 mM $KP_i$ buffer at pH 7.5, with 150 mM NaCl and 0.05% DDM. $T_m$ for each condition was monitored every day. One condition is incubating the protein with 5 % DMSO and the other one is 0 % DMSO. The percentage of DMSO in the wells during the TSA will be set to 2.5 %. The final ROX and GloMelt concentrations were set to 0.5 µM and 1x, respectively. Figure S8 depicts the corresponding curves for the ECF-PanT protein, illustrating the first derivative of fluorescence emission as a function of temperature. The first derivative of fluorescence emission provides valuable insights into the thermal stability characteristics of the proteins under investigation. (Huynh & Partch, 2015)

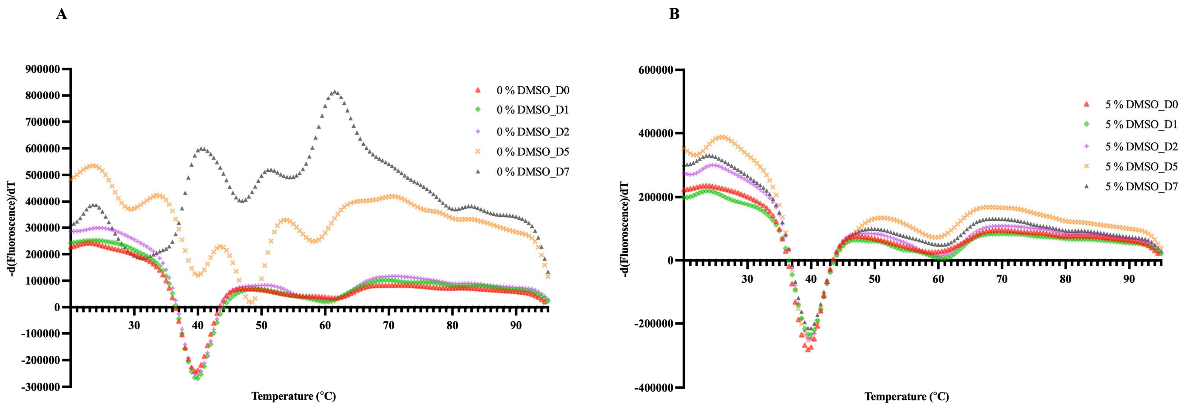


**Figure S8.** Corresponding ECF-PanT protein curves with A) 0% and B) 5% of DMSO.

In Figure S8-A, it can be observed that ECF-PanT, when incubated in the optimal buffer without DMSO (0% DMSO), remains stable for 3 days. However, beyond this period, the protein exhibits multiple melting temperatures, suggesting a loss of stability. Although a peak with approximately $T_m$ of 39.66 ºC is visible, it cannot be definitively concluded that the protein remains stable under these conditions. On the other hand, Figure S8-B illustrates the stability of ECF-PanT in the respective buffer containing 5% DMSO. In this case, the protein remains stable throughout the incubation period, indicating that the presence of 5% DMSO helps to maintain its stability. The small variation between the starting intensities could be due to an error in pipetting which could cause a difference in concentration. In Figures S9 and S10, the corresponding curves for ECF-module and ECF-FolT2 are presented, respectively. These curves provide insights into the stability profiles of these proteins under the specified experimental conditions.

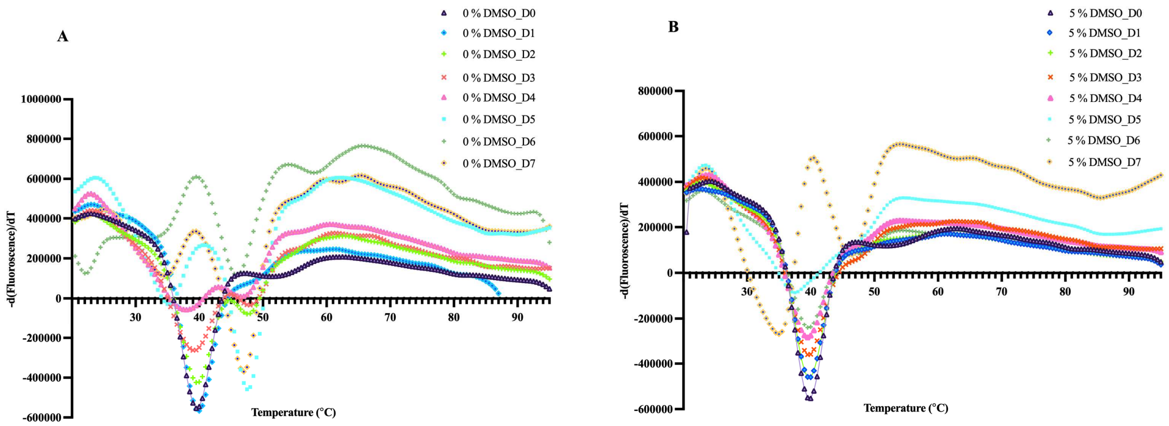


**Figure S9.** Corresponding ECF-module protein curves with A) 0% and B) 5% of DMSO.

In Figure S9-A, it is evident that the ECF-module with approx. $T_m$ of 39.45 °C, when incubated in the buffer without DMSO (0% DMSO), remains stable for 3 days. This indicates that the protein maintains structural integrity and stability during this timeframe. On the other hand, in Figure S9-B, when the ECF-module is incubated in the buffer containing 5% DMSO, it exhibits stability for an extended period of 3-4 days.

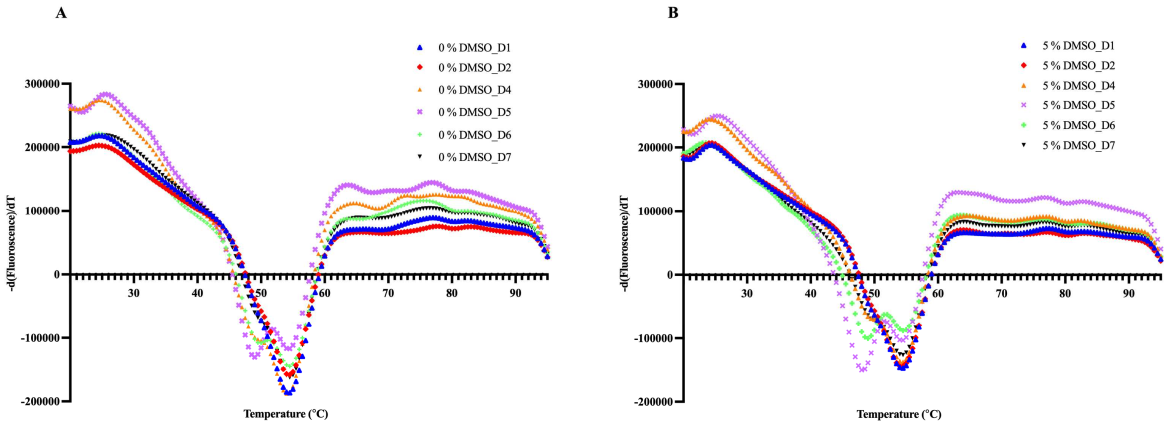


**Figure S10.** Corresponding ECF-FolT2 protein curves with A) 0% and B) 5% of DMSO.

Figure S9 shows that ECF-FolT2 is stable for 4 days with $T_m$ of approx. 54.57 °C, and after that, the curve shape and the fluorescence intensity start to change in both 0% and 5% of DMSO. The comparison between the two figures highlights the influence of DMSO on the stability of the ECF proteins, with the addition of 5% DMSO providing an advantage in terms of prolonging the protein's stability.

## Evaluation of stability data resulted from TSA

To evaluate the stability of ECF-PanT, we incubated the protein at room temperature in the optimal buffer (50 mM KPi pH 7.5, 50 mM NaCl, 0.05% DDM) under two conditions: with and without 5% DMSO. The stability was monitored by analyzing UV absorption at 280 nm using Size Exclusion Chromatography (SEC) to ensure consistent absorbance (see Figure 2B). Technical duplicates were used to confirm the reliability of the results. Following SEC, fractions were further analyzed by Sodium Dodecyl Sulfate-Polyacrylamide Gel Electrophoresis (SDS-PAGE) to verify the presence and integrity of the protein bands. The results are shown in Figure S11, confirming the expected bands and demonstrating the protein's stability.

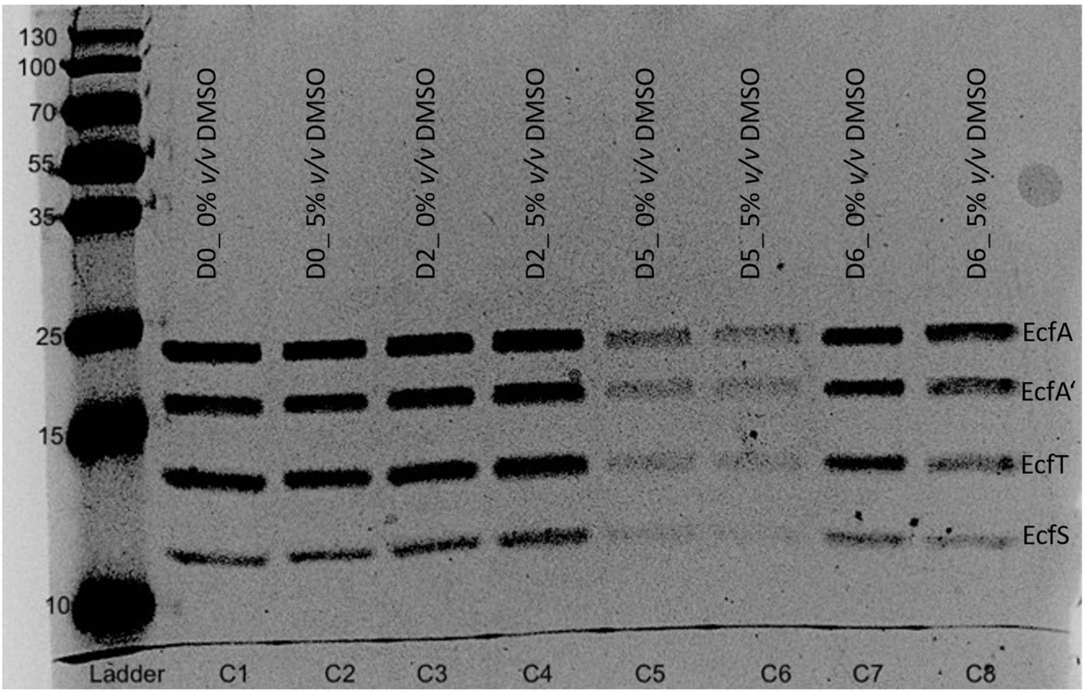


**Figure S11. SEC Fractions on SDS-PAGE (12% Gel).** Each condition indicated by **C** can be described respectively as **C1**) Day 0 (D0) without DMSO (0%). **C2**) Day 0 (D0) with 5% DMSO. **C3**) Day 2 (D2) without DMSO (0%). **C4**) Day 2 (D2) with 5% DMSO. **C5**) Day 5 (D5) without DMSO (0%). **C6**) Day 5 (D5) with 5% DMSO. **C7**) Day 6 (D6) without DMSO (0%). **C8**) Day 6 (D6) with 5% DMSO.

As depicted in **Figure S11**, all four bands corresponding to the protein of interest were observed throughout the experimental period. However, it is noteworthy that on day 5, the protein volume injection was lower compared to the other days. The presence of all four bands confirms the persistence of the protein fragments.

## Protein–ligand interaction study using TSA

The experimental setup, including run parameters, total volume, protein and dye concentrations, as well as the selection of the optimal buffer, remained consistent with previous procedures. All the compounds were diluted in 100% DMSO and for each compound, a specific range of concentration was chosen. The volumes for each component were 10 µL of protein, 2.5 µL of ROX, 2 µL of GloMelt, 1µL of the compound in 100% DMSO (or 100% DMSO only as control), and 4.5 µL of the buffer.

## Surface plasmon resonance (SPR)

The SPR binding studies were performed using a Reichert 4SPR surface plasmon resonance spectrometer (Reichert Technologies, Buffalo, NY, USA), and medium–density NTA derivatized polycarboxylate hydrogel NiHC200M sensor chips (XanTec Bioanalytics, Düsseldorf, Germany). The experimental procedure involved two rounds of nickel activation and one immobilization step at a concentration of 200 nM. The proteins ECF-module (92,56146 kDa), ECF-PanT (113,8745 kDa), and ECF-FolT2 (112,16316 kDa) were immobilized in flow cells 1, 2, and 3, respectively according to the standard protocol as provided by Xantec bioanalytics with a slight modification based on the recombinant proteins. The flow-cell 4 was left blank to serve as a reference.

The immobilization buffer employed for the immobilization process consisted of 10 mM HEPES with a pH of 7.5, 50 mM NaCl, 50 µM EDTA, and 0.05% DDM without DMSO. Following the immobilization step, for the binding study of the compounds, the running buffer was exchanged for the mentioned buffer with 5% *v/v* DMSO. This was done to ensure consistency in the percentage of DMSO present in the solutions containing the respective testing inhibitors, as these inhibitors were soluble in the DMSO solvent. By maintaining the same DMSO concentration, we aimed to minimize any potential solvent-related effects on the assay results. All running buffers were filtered and degassed before use.

The system was initially primed with 5 mM $NiCl_2$ with an association and dissociation time of 2 min for each to activate the NTA sensor chip. Then, the recombinant proteins with a concentration of 200 nM were injected at a flow rate of 10 µL/min for 8 min. Finally, the baseline check with the running buffer showed a stable immobilization level of approximately 9.000 RU, 14.000 RU, and 6.000 RU for the ECF-module, ECF-PanT, and ECF-FolT2, respectively.

First, the calibration curve for correction of the DMSO effect was prepared; the concentrations were 3%, 3.5% 4%, 4.5%, 5%, 5.5%, 6%, 6.5%, and 7% *v/v* and were diluted with SPR buffer. Then, the concentration of compounds started from 800 µM to 1.56 µM with a dilution factor of 2. The concentration of DMSO in all the samples was set to 5% *v/v* DMSO. The samples (200 µL) were loaded on a 96-well plate and were injected at a flow rate of 50 µL/min. Single-cycle kinetics were applied for $K_D$ determination. The association time was set to 60 s, and the dissociation phase was recorded for 120 s. For surface regeneration, 0.35 M EDTA (pH 8.5) and 0.1 M NaOH were used. Data processing and analysis were performed by Scrubber software (Version 2.0c, 2008, BioLogic Software). Sensorgrams were calculated by sequential subtractions of the corresponding curves obtained from the reference flow cell and the running buffer (blank). SPR responses were expressed in the resonance unit (RU). The $K_D$ values were calculated by global fitting of the kinetic curves as well as fitting of the steady state binding responses to a 1:1 Langmuir interaction model. The calculated values are in the same range.

The SPR experiments were conducted at least two times, representing independent replicates. The response-time and response-concentration curves for each compound, illustrating the binding interactions, are presented in Figure S12 for ECF-PanT, Figure S13 for ECF-FolT2, and Figure S14 for ECF-module. These curves provide insights into the kinetics and concentration-dependent responses of compounds **1–6** when interacting with the immobilized proteins.

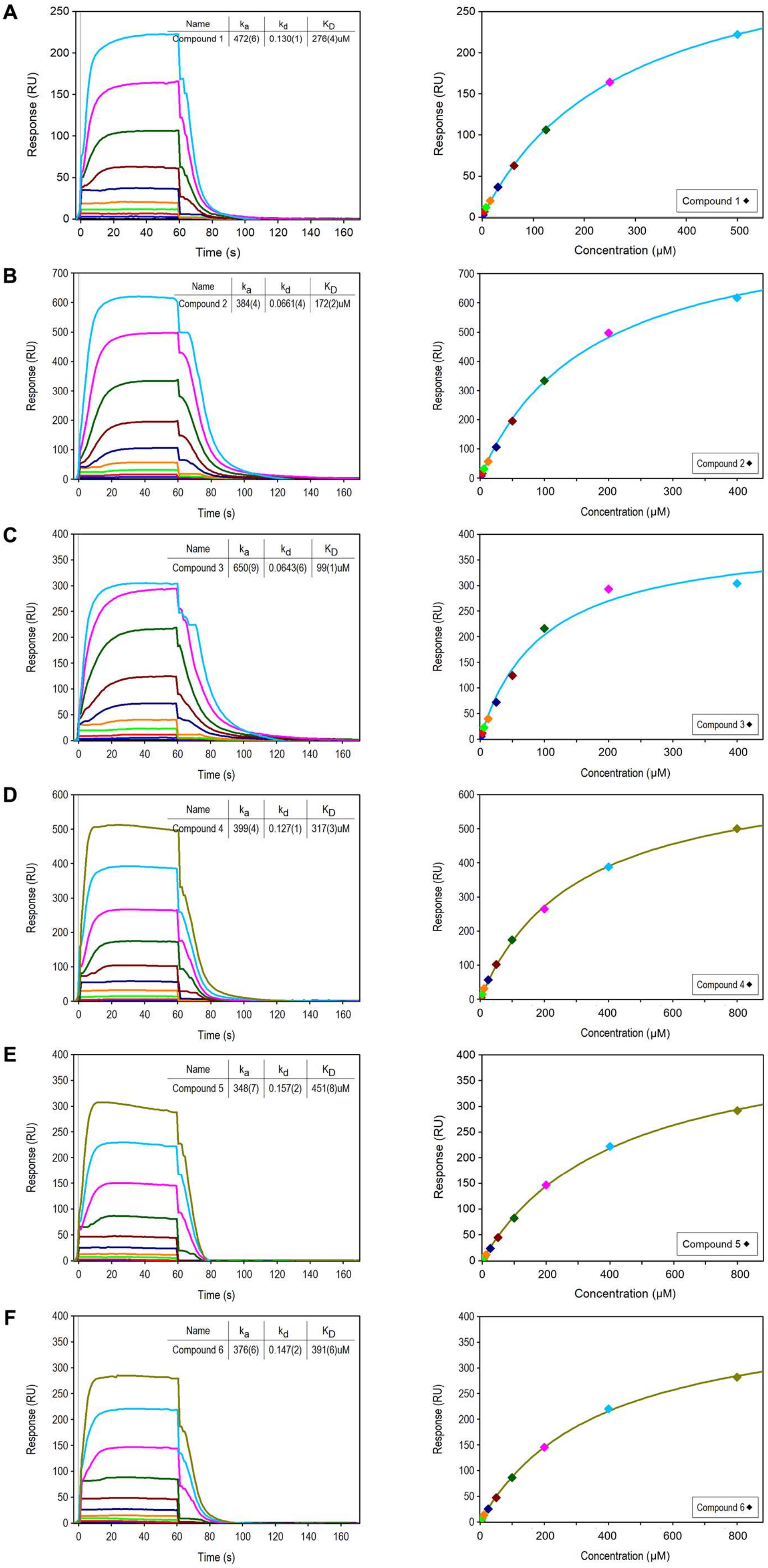


**Figure S12.** Response–time sensorgram overlay and response–concentration curves for the ECF-PanT protein upon injection of compounds **1**–**6**, denoted as A to F, respectively. Each compound is associated with a unique curve, elucidating the temporal evolution of the ECF-PanT protein's response as well as its response at different compound concentrations.

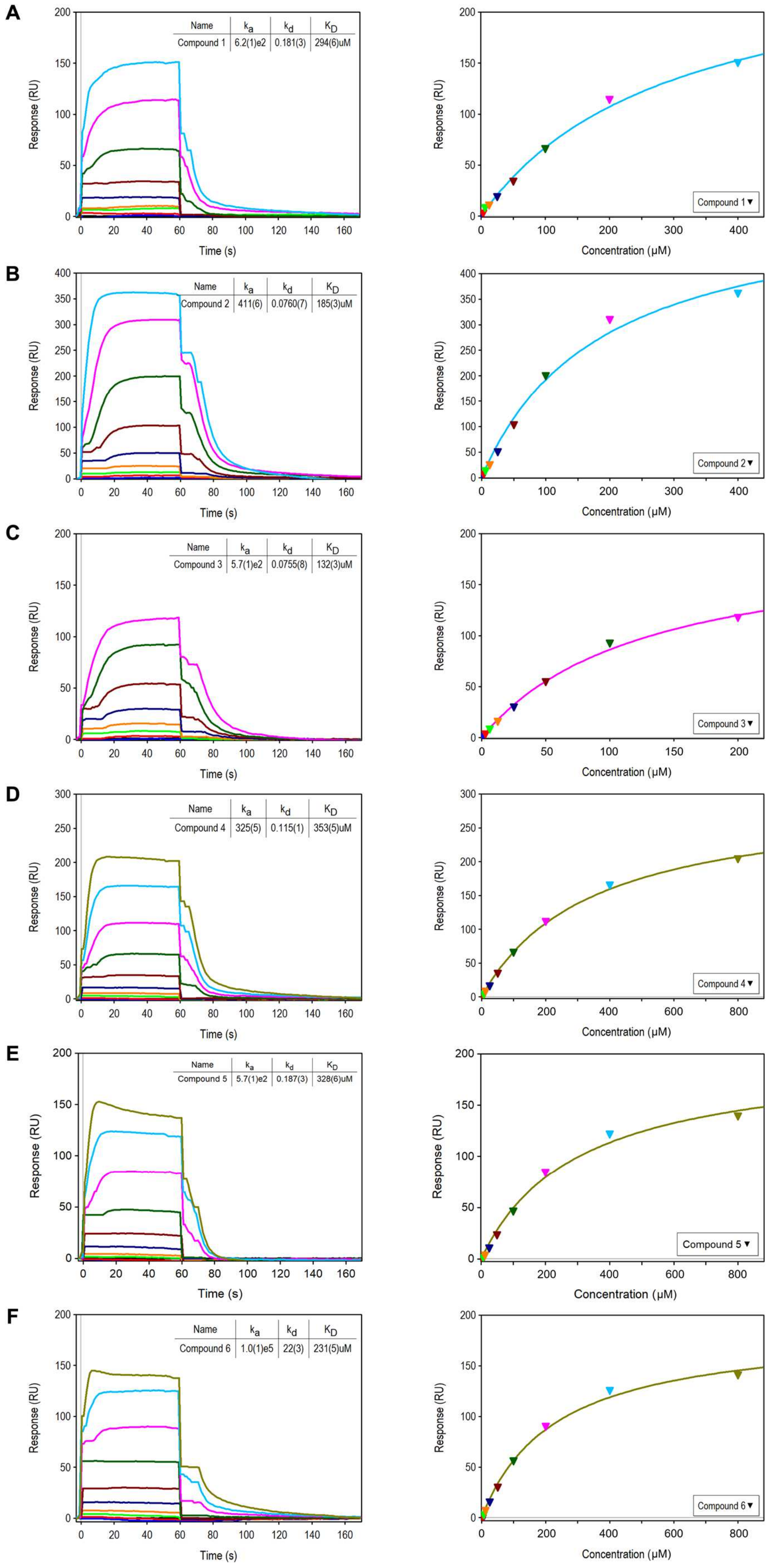


**Figure S13.** Response–time sensorgram overlay and response–concentration curves for the ECF-FolT2 protein upon injection of compounds **1**–**6**, denoted as A to F, respectively. Each compound is associated with a unique curve, elucidating the temporal evolution of the ECF-FolT2 protein's response as well as its response at different compound concentrations.

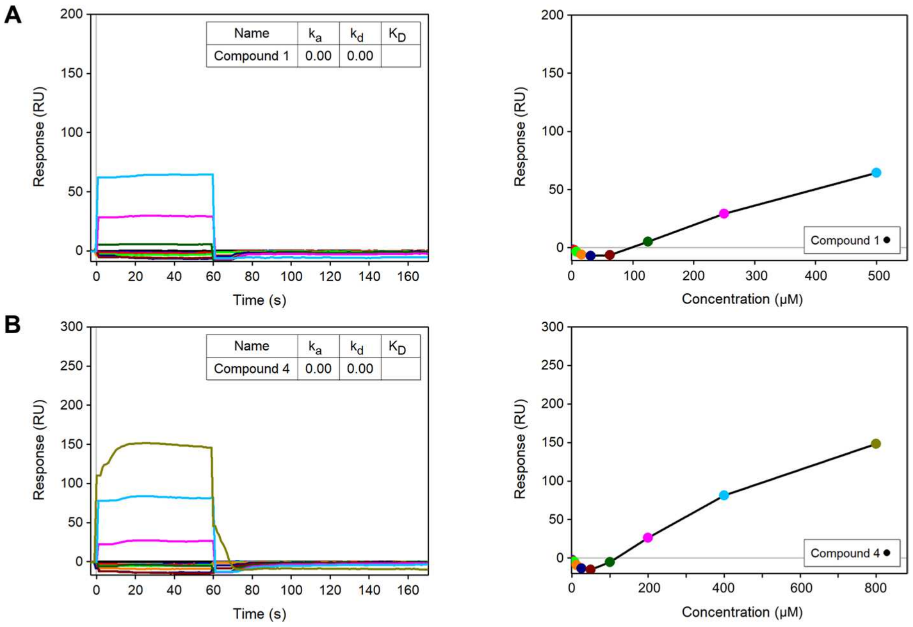


**Figure S14.** Response–time sensorgram overlay and response–concentration curves for the ECF-module upon injection of compounds **1** and **4** as representative of the two classes, denoted as A and B, respectively.

## In vitro cytotoxicity evaluation

As previously noted, this assay was conducted following the methodology of Haupenthal et al., 2007, with minor modifications, which will be detailed below.

To obtain information regarding the toxicity of our compounds, their impact on the viability of human cells was investigated. HepG2 and A549 cells ($2 \times 10^4$ cells per well) were seeded in 96-well, flat-bottomed culture plates in 100 µL culture medium (DMEM containing 10% fetal calve serum, 1% penicillin-streptomycin). Twenty-four hours after seeding the cells, medium was removed and replaced by medium containing test compounds in a final DMSO concentration of 1%. Compounds were tested in duplicates at a single concentration in 1% DMSO/medium. Epirubicin and doxorubicin were used as positive controls in serial dilutions starting from 10 µM, and rifampicin was used as a negative control (at 100 µM). The living cell mass was determined 48 h after treatment with compounds by adding 0.1 volumes of 3-(4,5-dimethylthiazol-2-yl)-2,5-diphenyltetrazolium bromide (MTT) solution (5 mg/mL sterile PBS) (Sigma, St. Louis, MO) to the wells. After incubating the cells for 30 min at 37 °C (atmosphere containing 5% $CO_2$), medium was removed and MTT crystals were dissolved in 75 µL of a solution containing 10% SDS and 0.5% acetic acid in DMSO. The optical density (OD) of the samples was determined photometrically at 570 nm in a PHERAstar Omega plate reader (BMG labtech, Ortenberg, Germany). To obtain percent viability for each sample, their ODs were related to those of DMSO controls. At least two independent measurements were performed for each compound. The calculation of $IC_{50}$ was performed using the nonlinear regression function of GraphPad Prism 10 (GraphPad Software, San Diego, CA, USA).

## References


Altschul SF, Madden TL, Schäffer AA, Zhang J, Zhang Z, Miller W, Lipman DJ (1997) Gapped BLAST and PSI-BLAST: a new generation of protein database search programs. Nucleic Acids Research, 25:3389–3402.

Altschul SF, Wootton JC, Gertz EM, Agarwala R, Morgulis A, Schäffer AA, Yu YK (2005) Protein database searches using compositionally adjusted substitution matrices. FEBS J 272:5101–5109.

Di Tommaso, P., Moretti, S., Xenarios, I., Orobitg, M., Montanyola, A., Chang, J. M., Notredame, C. (2011). T-Coffee: A web server for the multiple sequence alignment of protein and RNA sequences using structural information and homology extension. Nucleic Acids Research, 39(SUPPL. 2).

Haupenthal J, Baehr C, Zeuzem S, Piiper A (2007) RNAse A-like enzymes in serum inhibit the anti-neoplastic activity of siRNA targeting polo-like kinase 1. Int. J. Cancer 121: 206–210.

Huynh, K., & Partch, C. L. (2015). Analysis of Protein Stability and Ligand Interactions by Thermal Shift Assay. (February), 1–14. doi: 10.1002/0471140864.ps2809s79

Notredame, C., Higgins, D. G., & Heringa, J. (2000). T-coffee: A novel method for fast and accurate multiple sequence alignment. Journal of Molecular Biology, 302(1), 205–217.

Robert, X, Gouet, P (2014) Deciphering key features in protein structures with the new ENDscript server. Nucleic Acids Research, 42:W320–W324. doi:10.1093/nar/gku316

Setyawati I, Stanek WK, Majsnerowska M, Swier LJYM, Pardon E, Steyaert J, Guskov A, Slotboom DJ (2020) In vitro reconstitution of dynamically interacting integral membrane subunits of energy-coupling factor transporters. Elife. 22;9:e64389. doi: 10.7554/eLife.64389.

Swier, L. J. Y. M., Guskov, A., & Slotboom, D. J. (2016). Structural insight in the toppling mechanism of an energy-coupling factor transporter. Nature Communications, 7.

Zhang M, Bao Z, Zhao Q, Guo H, Xu K, Wang C, Zhang P (2014) Structure of a pantothenate transporter and implications for ECF module sharing and energy coupling of group II ECF transporters. Proc Natl Acad Sci U S A 111:18560–18565.
